# Synthesis of Cyano-Benzylidene Xanthene Synthons Using a Diprotic Brønsted Acid Catalyst, and Their Application as Efficient Inhibitors of Aluminum Corrosion in Alkaline Solutions

**DOI:** 10.3390/molecules27175733

**Published:** 2022-09-05

**Authors:** Mohammed A. Amin, Gaber A. M. Mersal, Morad M. El-Hendawy, Abdallah A. Shaltout, Ali Badawi, Johan Boman, Adil A. Gobouri, Murat Saracoglu, Fatma Kandemirli, Rabah Boukherroub, Jacek Ryl, Mohamed E. Khalifa

**Affiliations:** 1 Department of Chemistry, College of Science, Taif University, P.O. Box 11099, Taif 21944, Saudi Arabia; 2 Chemistry Department, Faculty of Science, New Valley University, Kharga 72511, Egypt; 3 Department of Physics, College of Science, Taif University, P.O. Box 11099, Taif 21944, Saudi Arabia; 4 Department of Chemistry and Molecular Biology, Atmospheric Science, University of Gothenburg, 412 96 Gothenburg, Sweden; 5 Faculty of Education, Erciyes University, 38039 Kayseri, Turkey; 6 Department of Biomedical Engineering, Faculty of Engineering and Architecture, Kastamonu University, 37150 Kastamonu, Turkey; 7 University of Lille, CNRS, Centrale Lille, University Polytechnique Hauts-de-France, UMR 8520-IEMN, F-59000 Lille, France; 8 Division of Electrochemistry and Surface Physical Chemistry, Institute of Nanotechnology and Materials Engineering, Gdansk University of Technology, Narutowicza 11/12, 80-233 Gdansk, Poland

**Keywords:** xanthene dyes, dimedone, aluminum corrosion, inhibition, DFT, Monte Carlo simulations

## Abstract

Novel cyano-benzylidene xanthene derivatives were synthesized using one-pot and condensation reactions. A diprotic Brønsted acid (i.e., oxalic acid) was used as an effective catalyst for the promotion of the synthesis process of the new starting xanthene–aldehyde compound. Different xanthene concentrations (ca. 0.1–2.0 mM) were applied as corrosion inhibitors to control the alkaline uniform corrosion of aluminum. Measurements were conducted in 1.0 M NaOH solution using Tafel extrapolation and linear polarization resistance (LPR) methods. The investigated xanthenes acted as mixed-type inhibitors that primarily affect the anodic process. Their inhibition efficiency values were enhanced with inhibitor concentration, and varied according to their chemical structures. At a concentration of 2.0 mM, the best-performing studied xanthene derivative recorded maximum inhibition efficiency values of 98.9% (calculated via the Tafel extrapolation method) and 98.4% (estimated via the LPR method). Scanning electron microscopy (SEM) was used to examine the morphology of the corroded and inhibited aluminum surfaces, revealing strong inhibitory action of each studied compound. High-resolution X-ray photoelectron spectroscopy (XPS) profiles validated the inhibitor compounds’ adsorption on the Al surface. Density functional theory (DFT) and Monte Carlo simulations were applied to investigate the distinction of the anticorrosive behavior among the studied xanthenes toward the Al (111) surface. The non-planarity of xanthenes and the presence of the nitrile group were the key players in the adsorption process. A match between the experimental and theoretical findings was evidenced.

## 1. Introduction

Aluminum (Al), one of the most widely used metals worldwide, has a low density, good appearance, and outstanding electrical and thermal conductivity. It is relatively non-precious, and almost twice as plentiful as iron. Some Al alloys’ high strength-to-weight ratio is also equivalent to that of high-strength structural steels. In addition, despite being a highly active metal (a powerful reducing agent with a standard reduction potential of *E*°_Al3+/Al_ = −1.662 V), metallic Al is immune to corrosion due to the instantaneous formation of a stable, virtually inert compact passive oxide film on its surface [1]. This passive film creates a barrier protecting the base metal from corrosion. Because of this combination of featured properties, Al and its alloys have become a preferred option for numerous industrial applications, such as cars and airplanes, electronic equipment, construction materials, etc. [1,2,3,4].

Despite Al’s tremendous advantages relative to other metals, it is not always fully resistant to corrosion. The protective oxide film instantaneously formed on Al only resists corrosion properly in aqueous media with a narrow pH range of 4.0 to 7.5, but dissolves significantly upon exposure to strongly acidic or alkaline electrolytes, leaving the base metal at risk of corrosion [5,6,7].

One of the major problems of corrosion of Al and its alloys is encountered in energy storage devices (e.g., batteries), where it several problems, such as passivation of the active cathode material and formation of solid corrosion products, resulting in enhanced electrical resistance. The electrolyte is also polluted by the Al’s soluble corrosion products, and the rate of self-discharge is increased accordingly [8,9]. For these reasons, several researchers have dedicated their efforts to studying and mitigating the corrosion of Al and Al alloys in alkaline solutions—the most common electrolytes employed in Al–air batteries [10,11].

The use of inhibitors is one of the best-known methods for the mitigation of metal corrosion. since such an approach requires no special equipment; it is safe, non-precious, and easy to operate [12]. Corrosion inhibitors limit the undesirable damaging effects of corrosion effectively and, therefore, significantly reduce the rate of metal dissolution [12]. It is therefore important to apply inhibitors to control the corrosion of metals and alloys that are in contact with aggressive environments.

Many organic compounds have been investigated and documented in the literature as effective corrosion inhibitors for Al and its alloys in various aggressive aqueous media [11,13]. The organic molecules are believed to prevent corrosion through adsorption at the metal–solution interface. The fundamental factors controlling the inhibitor molecules’ adsorption are the chemical structure of the inhibitor molecule, the environment, and the nature of the metal surface [11,12,13,14]. A polar group such as (-CO_2_H) increases the inhibition performance by acting as a source of electrons, but the charge moving from or into the benzene-ring-containing compounds is less than one electronic charge, and is spread over the entire ring [12,14].

In hetero systems, the metal’s electron density at the point of contact with the adsorbed inhibitor molecule is altered depending on whether the inhibitor molecule’s ring is attached to the metal surface via the S or N atom. This, in turn, slows down the cathodic or anodic reaction as electrons are devoured at the cathode and provided at the anode [15]. Effective organic inhibitors have the tendency to donate electrons to the metal surface’s vacant d-orbitals to form coordinate covalent bonds, and at the same time, using their anti-bonding orbitals, accept the metal surface’s free electrons to form feedback bonds to further strengthen adsorption [11,12,13,14].

Xanthenes (dibenzo[*a*,*e*]pyrans) are tricyclic molecules containing a pyran heterocycle as the central ring, fused on both sides to benzene rings. They are commonly synthesized using Lewis acid catalysts in the presence and absence of solvents [16]. Xanthene dyes have been found to be useful in recent years because of their fundamental role in diverse biochemical and industrial fields [17,18,19,20,21,22,23]. Multicomponent processes have recently gained significant economic and ecological importance in the mainstream of current interest, as they have been shown to be a very elegant and rapid way of producing complex structures from basic building blocks in a single synthetic process, and display high atomic economy and high selectivity [24].

This prompted us to design and synthesize a new generation of xanthene dyes via multicomponent methods and apply them as efficient aluminum corrosion inhibitors. The inhibitive effect is believed to occur through the donation of p-electrons along with unshared p-electrons of N atoms to induce greater adsorption of the inhibitor molecule onto the surface of Al in alkaline media.

## 2. Materials and Methods

### 2.1. Chemicals and Reagents

All of the chemicals, reagents, and solvents were chemically pure and purchased from Sigma-Aldrich Chemicals Company (Burlington, MA, USA).

### 2.2. Instrumentation

The melting points of the synthesized compounds were determined in open-glass capillaries on a Stuart SMP20 melting point apparatus (Bibby Scientific Limited, Staffordshire, UK), and were uncorrected. Elemental analyses (C, H, N) were conducted using the PerkinElmer 2400 Analyzer, series II (PerkinElmer Co., Shelton, UK); their results were found to be in good agreement (±0.3%) with the calculated values. The infrared spectra were recorded on a PerkinElmer Alpha Platinum-ATR spectrometer, and the NMR spectra were measured on a Bruker WP 300 (Bruker, Billerica, MA, USA) in DMSO, using TMS (Me_4_Si) as an internal standard. The ^1^H- and ^13^C-NMR chemical shifts were reported as parts per million (ppm) downfield from TMS. The splitting patterns are designated as follows: s, singlet; d, doublet; m, multiplet. Mass spectra were acquired on a GC-MS spectrometer (Shimadzu Qp-2010 Plus, Kyoto, Japan). All of the microanalyses and spectral analyses were performed at the Micro Analytical Centers of Taif University (C, H, N, and IR spectra), King Abdel-Aziz University (^1^H- and ^13^C-NMR analysis), and Cairo University (Mass Spectra).

### 2.3. Electrochemical Measurements

An ultrapure Al wire (Sigma-Aldrich, 1.0 mm diameter, 99.999%, 266558-10.5G, 01311LB, CAS 7429-90-5; FW 26.98; MW05152, Burlington, MA, USA) was used in this work as the working electrode (WE). The pure Al wire was firmly surrounded by a Teflon jacket for electrochemical tests, leaving a constant surface area of 0.00785 cm^2^ in contact with the test solution. For surface characterizations, 0.1 mm thick Al sheets (1 cm × 2 cm rectangular coupons) of the same purity were used. Before each run, the investigated specimens (both the Al sheets and the Al wire) were subjected to mechanical abrasion using a number of emery papers of up to 1200 grade. Then, prior to submerging the abraded specimen in the test solution, it was scrubbed in acetone and double-distilled water. The employed chemicals’ solutions were freshly prepared using Millipore Milli-Q filtered water.

Electrochemical measurements were conducted in naturally aerated 1.0 M NaOH solutions devoid of and containing different concentrations (0.1–10 mM) of the newly synthesized xanthene derivatives, namely, compounds (**3**), (**4**), (**5**), and (**6**). A newly prepared solution was used for each run, as well as a cleaned collection of electrodes. The temperature of the test solution was maintained constant at 25 ± 1 °C using a water thermostat. Electrochemical characterizations were carried out in a standard (double-jacketed) electrochemical cell with a large inner volume of 200 mL, using a Pt wire as the auxiliary electrode. The reference electrode was Ag/AgCl, inserted in a Luggin capillary with its tip placed very close to the working electrode surface to reduce the IR drop. The electrochemical cell was connected to an Autolab Potentiostat/Galvanostat (PGSTAT30).

The electrochemical techniques employed to monitor the uniform corrosion rate of Al and to evaluate the inhibition performance of the investigated organic compounds, as well as to gain more insight into the inhibition mechanism, were Tafel extrapolation and linear polarization resistance (LPR). Prior to conducting the LPR run, the working electrode was first subjected to 3 h of stabilization at the rest potential in the test solution. This duration was quite adequate for the rest potential to achieve a quasi-stationary value.

After this WE’s stabilization period, the LPR measurements were conducted by sweeping the WE’s potential from −20 to +20 mV vs. the corrosion potential (*E*_corr_), at a scan rate of 0.2 mV s^−1^. After the LPR run was completed, linear sweep voltammetry (LSV) measurements were performed by polarizing the WE, starting from a cathodic potential of *E*_corr_ − 0.25 V vs. Ag/AgCl to an anodic potential of *E*_corr_ + 0.25 V vs. Ag/AgCl, at a 1.0 mV s^−1^ sweep rate.

In order to ensure that the findings were reproducible, at least three different tests were conducted for each run. The results’ reproducibility was found to be good. The average values (standard deviation) of the various electrochemical kinetic parameters derived from the three performed independent experiments are reported here.

The morphology of corroded and inhibited Al surfaces was assessed using a JEOL JSM 6390 LA Analytical Scanning Electron Microscope (JEOL, Tokyo, Japan). The compositions of such surfaces were determined using ZAF software to quantify the energy-dispersive X-ray spectroscopy (EDS) spectra obtained via an EDS attachment (JEOL EDS EX-54175JMU) on the JEOL SEM.

### 2.4. Computational Details

#### 2.4.1. Quantum Chemical Calculations on Isolated Molecules

The calculations were performed using B3LYP/6-311++G(2d,2p)//B3LYP/6-311(d,p) model chemistry as implemented in Gaussian 16 code [25]. Full geometric optimizations of the isolated molecules were carried out in the water phase using the universal solvation model (SMD) [26].

#### 2.4.2. Monte Carlo Simulations

The adsorption process of the titled compounds on the aluminum surface was investigated by performing Metropolis Monte Carlo simulations [27] using the adsorption locator module, as implemented in Materials Studio 2017 [28]. Because Al (111) is the most stable of all Al facets [29], it was selected to simulate the adsorption process. The size of the simulation box was 2.9 × 2.9 × 5.9 nm. The interaction between the studied inhibitors and the Al (111) surface was carried out under periodic boundary conditions. The metallic surface was composed of four layers of Al atoms to ensure sufficient depth, where each layer included 121 atoms. The COMPASS force field [30] was employed for the conduction of simulations, where Ewald and atom-based summation approaches were used to calculate the electrostatic and van der Waals energetic components, respectively. To simulate the medium effect of 1.0 M NaOH, 9 NaOH molecules and 500 H_2_O molecules with 1 inhibitor molecule were loaded together on the aluminum surface.

## 3. Results and Discussion

### 3.1. Chemistry

**Scheme 1:**
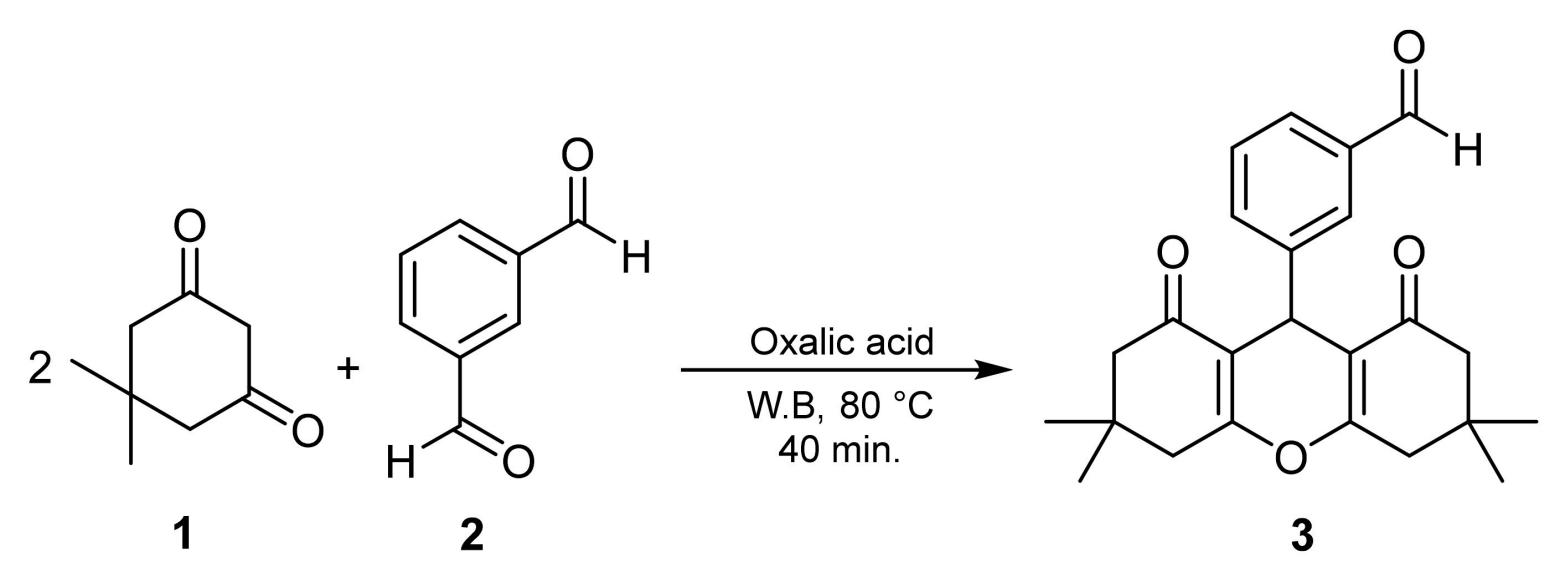
Synthesis of benzaldehyde xanthene derivative (**3**).

In accordance with our plan to develop effective organic corrosion inhibitors, it is of interest to produce a xanthene analog carrying hetero atoms of unshared electrons [16]. The delocalized p-electrons of benzene rings, along with unshared electrons of the polar patterns, could lead to enhancement of the inhibitor’s adsorption onto the metal surface (i.e., Al) [19]. The Brønsted diprotic oxalic acid efficiently catalyzed the synthesis of xanthene derivatives for the promotion of the one-pot multicomponent and condensation reactions [21]. Indeed, the domino reaction of two factors (i.e., 2 moles of dimedone (**1**) and 1 mole of isophthaldehyde (**2**)) was thus conducted in the presence of a catalytic amount of oxalic acid, yielding the benzaldehyde xanthene derivative (**3**), as shown in Scheme 1. The general procedure for the synthesis of the studied xanthene derivatives is presented in the Supplementary Materials (Section S1). The structure of compound (**3**) was elucidated by means of its spectral analysis, where an informative singlet signal appeared at *δ* = 9.88 ppm due to formyl hydrogen in the ^1^H-NMR spectrum (Supplementary Materials, Figure S1), in addition to the characteristic signals of xanthene analogs.

**Scheme 2:**
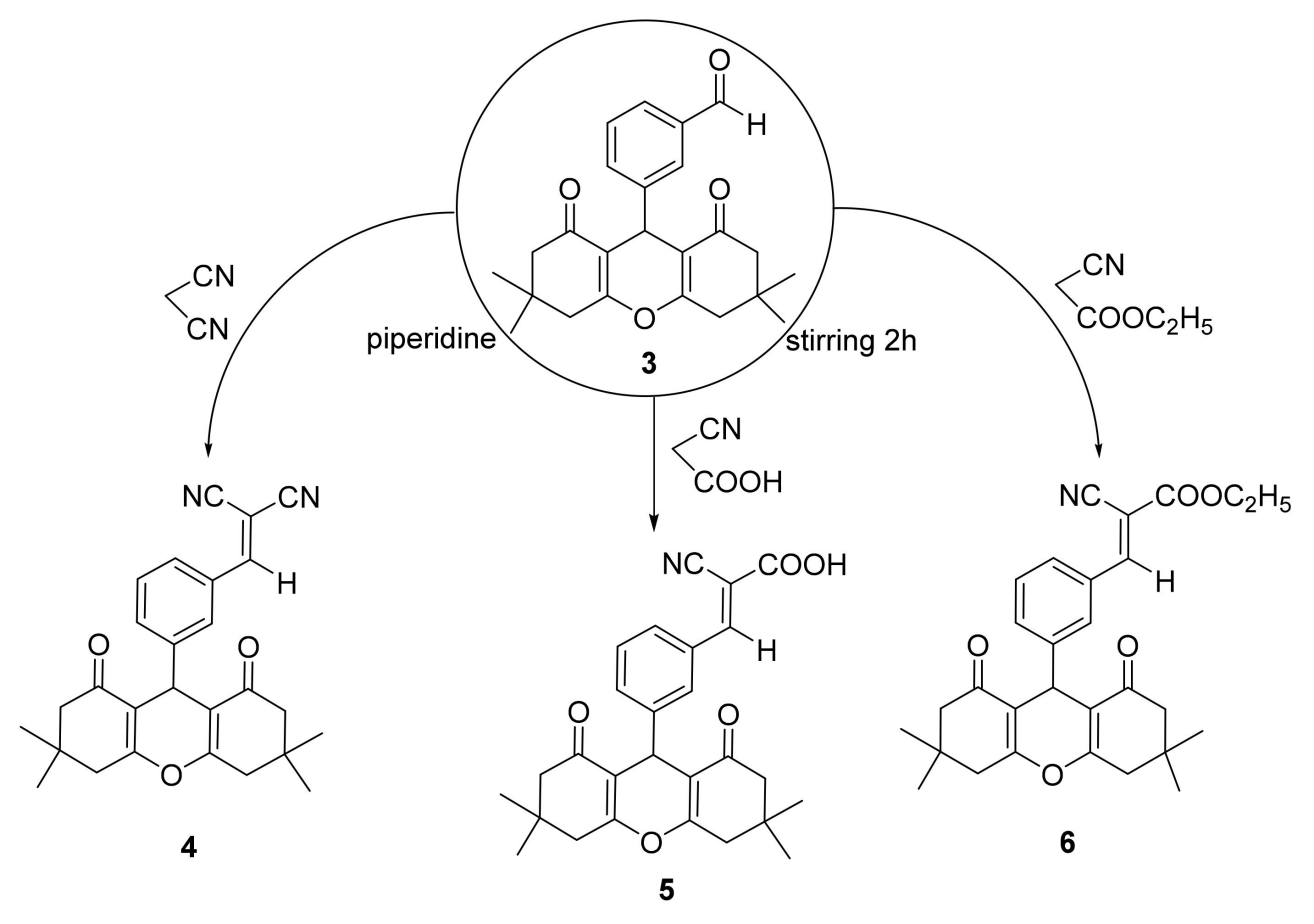
Synthesis of target xanthene derivatives (**4**)–(**6**).

The IR and ^1^H-NMR plots of xanthene synthons (**4**)–(**6**) (see Scheme 2) are depicted in Supplementary Materials, Figures S2–S7. The IR spectra featured characteristic sharp absorption bands at 2190–2232 cm^−1^ due to stretching vibrations of the cyano groups. Moreover, the ^1^H-NMR spectra displayed singlets at 7.93–8.53 ppm for the olefinic protons, in addition to a singlet at 12.45 ppm for the COOH group (compound **5**), and characteristic triplet (*δ* = 1.31) and quartet (*δ* = 4.25) signals for the methyl and methylene groups, respectively (compound **6**).

### 3.2. Polarization Studies

**Figure 1:**
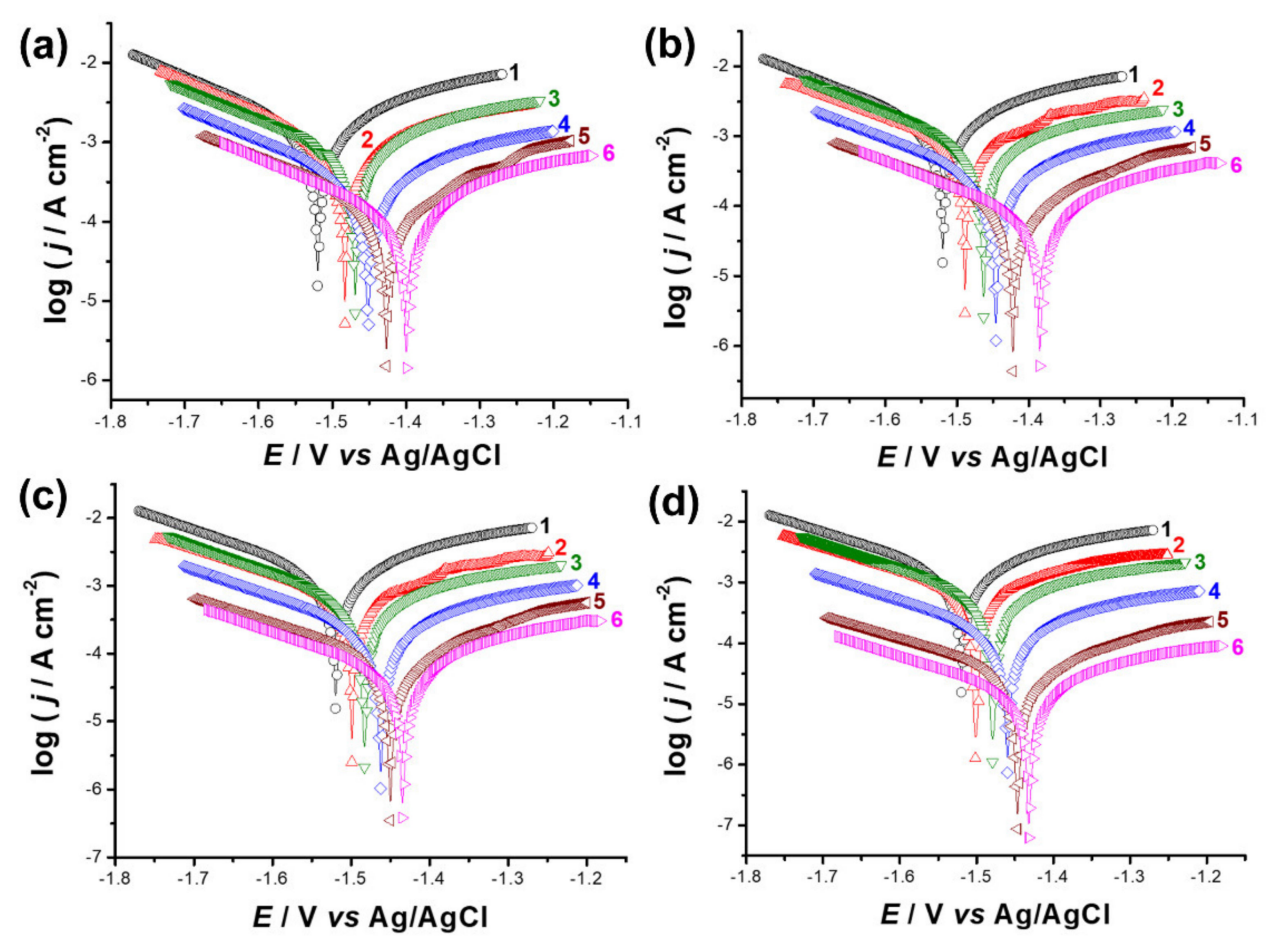
Cathodic and anodic polarization curves recorded for Al in 1.0 M NaOH solution devoid of and containing various concentrations (ca. 0.1–2.0 mM) of inhibitors: (**a**) (**3**), (**b**) (**4**), (**c**) (**5**), and (**d**) (**6**). Measurements were carried out at a scan rate of 5.0 mV s^−1^ at 25 °C. (1) 0.0 M, (2) 0.1 mM, (3) 0.2 mM, (4) 0.5 mM, (5) 1.0 mM, and (6) 2.0 mM inhibitor.

**Figure 2:**
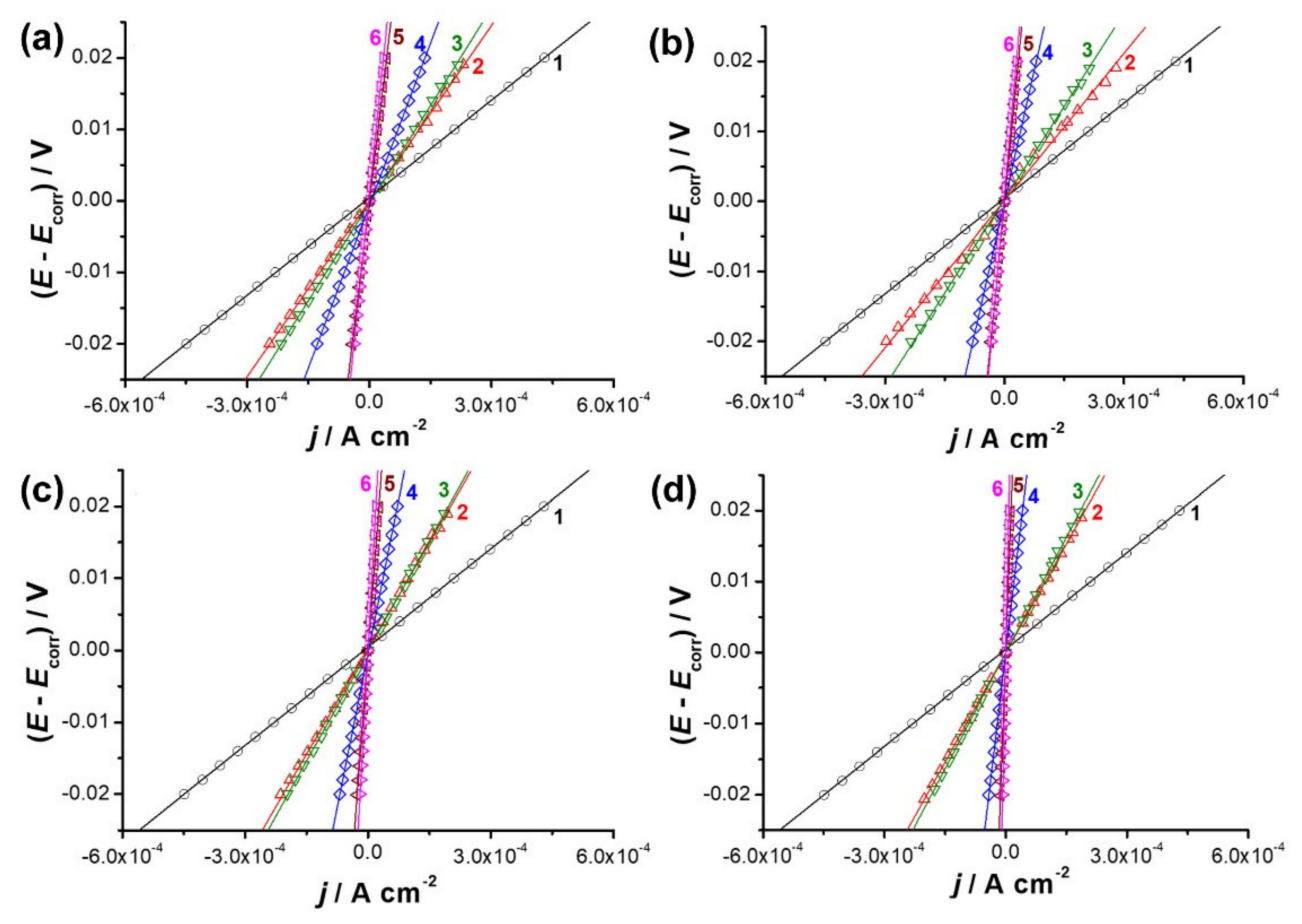
LPR plots constructed for Al in 1.0 M NaOH solution with and without different concentrations (ca. 0.1–2.0 mM) of inhibitors: (**a**) (**3**), (**b**) (**4**), (**c**) (**5**), and (**d**) (**6**). Measurements were carried out at a scan rate of 5.0 mV s^−1^ at 25 °C. (1) 0.0 M, (2) 0.1 mM, (3) 0.2 mM, (4) 0.5 mM, (5) 1.0 mM, and (6) 2.0 mM inhibitor.

Figure 1 and Figure 2 depict the LSV and LPR measurements recorded for Al in 1.0 M NaOH solution without (curve 1) and with (curves 2–6) different concentrations (0.1–10 mM) of the four tested xanthene derivatives.

Depending upon the type and concentration of the additive, the cathodic and anodic polarization branches of the current densities diminished, associated with a pronounced alternation in the location of *E*_corr_. In all cases, it was clear that the current associated with the anodic polarization curves was noticeably decreased upon introducing the inhibitor, whilst the cathodic curves were marginally skewed towards lower current densities. These findings indicate that the studied organic compounds inhibit the anodic processes more efficaciously than the cathodic ones. The tested xanthene derivatives can therefore be classified as mixed-type inhibitors with preponderant effects on the anodic reaction.

Further inspection of the cathodic and anodic polarization curves reveals that the cathodic branches exhibit a typical Tafel response, whereas the anodic branches lack this linear *E*-log *j* relationship—most likely due to the deposition of the corrosion products and/or passivation. A precise computation of the cathodic Tafel slope (*β*_c_) as well as the corrosion current density (*j*_corr_) by the Tafel extrapolation method is therefore feasible. The deficiency of Tafel behavior in the anodic domains, on the other hand, makes it difficult to obtain an accurate calculation of the anodic Tafel slope (*β*_a_) [31,32,33,34]. This is why the numerical values of *β*_a_ calculated from the software are not included here. 

Alternatively, accurate *β*_a_ values were obtained by constructing the calculated anodic Tafel line, following the work of McCafferty, who addressed the validity and drawbacks of the Tafel extrapolation method for precise evaluation of the corrosion rates in activation-controlled corrosion processes [35]. It was reported in McCafferty’s work that, in the Tafel extrapolation method, the use of both the anodic and cathodic Tafel regions is certainly favored over the use of just one Tafel region. On the experimental anodic polarization data, some mathematical treatments were performed on the basis of the work of McCafferty [35] to establish the calculated anodic Tafel line, as follows: the Tafel region in the cathodic polarization curve was first extrapolated to potential values beyond *E*_corr_, and then the anodic current density, *j*_a_, was calculated using Equation (1) [35]:

*J*_a_ (net experimental) = *j*_a_ − |*j*_c_| (1)

In Equation (1), the subscripts “a” and “c” refer to the anodic and the cathodic directions, respectively, while |*jc*| is the absolute cathodic current density value. The estimated anodic current density, *j*_a_, is therefore the amount of the anodic current density observed experimentally and the extrapolated cathodic current density [35]. The value of *β*_a_ can then be determined accurately by extrapolating the calculated anodic Tafel line back to *E*_corr_. The LPR lines’ slopes (see Figure 2) define the polarization resistance (*R*_p_). The various electrochemical kinetic parameters derived from such polarization measurements, based on the performed mathematical analyses, are shown in Supplementary Materials (Section S3, Tables S1–S4).

The Tafel slopes (*β*_a_ and *β*_c_) and *R*_p_ values can be inserted into the Stern–Geary equation [36] (Equation (2)) to determine the values of *j*_corr_ and, thus, the rates of corrosion, with high accuracy.

*j*_corr_ = *B*/*R*_p_ = {*β*_a_ *β*_c_/2.303(*β*_a_ + *β*_c_)}/*R*_p_ (2)

The values of *j*_corr_ (derived from the methods of Tafel extrapolation and LPR) can be used to calculate the inhibition efficiency value, *I*(%) (Tables S1–S4), for each studied inhibitor as a function of its concentration, using Equation (3):

{(*j*^o^_corr_ − *j*_corr_)/*j*^o^_corr_} × 100 (3)

where *j*^o^_corr_ and *R*_o_ are the corrosion current density and the polarization resistance recorded in the uninhibited solutions, respectively. Those measured in the inhibited solutions are termed *j*_corr_ and *R*_i_, respectively.

Equation (4) is employed to convert the values of *j*_corr_ (measured in µA cm^−2^) into corrosion rate, υ (expressed in mm/year) [37], as shown in Tables S1–S4.

υ = *K* × (*j*_corr_/*ρ*) × *EW* (4)

where *K* is a constant (3.27 × 10^−3^ mm g/µA cm yr), and *EW* and *ρ* are the Al’s equivalent weight (8.994 g) and density (2.7 g cm^−3^), respectively.

It is clear that for any tested inhibitor the *j*_corr_ values and, hence, the corrosion rate (υ), decrease (corresponding to enhanced *R*_p_ values) upon increasing the *C*_inhib_. These events occur to an extent dependent on the chemical structure of the investigated inhibitor. The decrease in *j*_corr_ and υ values, and the subsequent increase in the values of *R*_p_, generally indicates a mitigated corrosion rate, corresponding to ameliorated inhibition performance.

At any *C*_inhib_ value, the order of the rate decrease of uniform Al corrosion was found to be (**3**) > (**5**) > (**6**) > (**4**). This corresponds to an obvious increase in the values of *R*_p_ and *I* (%), following the sequence (**3**) < (**5**) < (**6**) < (**4**). These findings reveal the high inhibition performance of derivative (**4**) towards the alkaline corrosion of Al. Further inspection of Tables S1–S4 indicates that the Tafel slopes are greater than expected. For example, Tafel slope values of −249 and +241 mV dec^−1^ were recorded for the cathodic (i.e., the reduction of both water molecules and dissolved O_2_ occurring on its surface) and anodic (i.e., metal dissolution) processes, respectively, on Al in 1.0 M NaOH solutions without the inhibitor. No significant changes were observed for the value of *β*_c_ in the presence of increased concentrations of any of the four studied inhibitors. Metikos-Hukovic et al. [38] recorded large *β*_c_ values ranging from 235 to 245 mV dec^−1^ for the mitigation of Al corrosion in perchloric acid solutions using certain organic additives.

Since high Tafel slope values are often regarded as irregular, they cannot be applied for any mechanism prediction [38]. Metikos-Hukovic et al. [38] related the higher Tafel slope values to the Al passivity that occurs instantaneously, creating a virtually stable ineffective passive layer that limits the Al’s reduction potential [39]. This, in turn, delays any reduction at the surface by affecting the energy of the double-layer reaction, adding a barrier to the charge transfer through the film, or both [40]. The barrier-film model for the observed HER [40] was a consistent way of explaining the high Tafel slopes.

### 3.3. Characterization of the Corroded and Inhibited Al Surfaces

**Figure 3:**
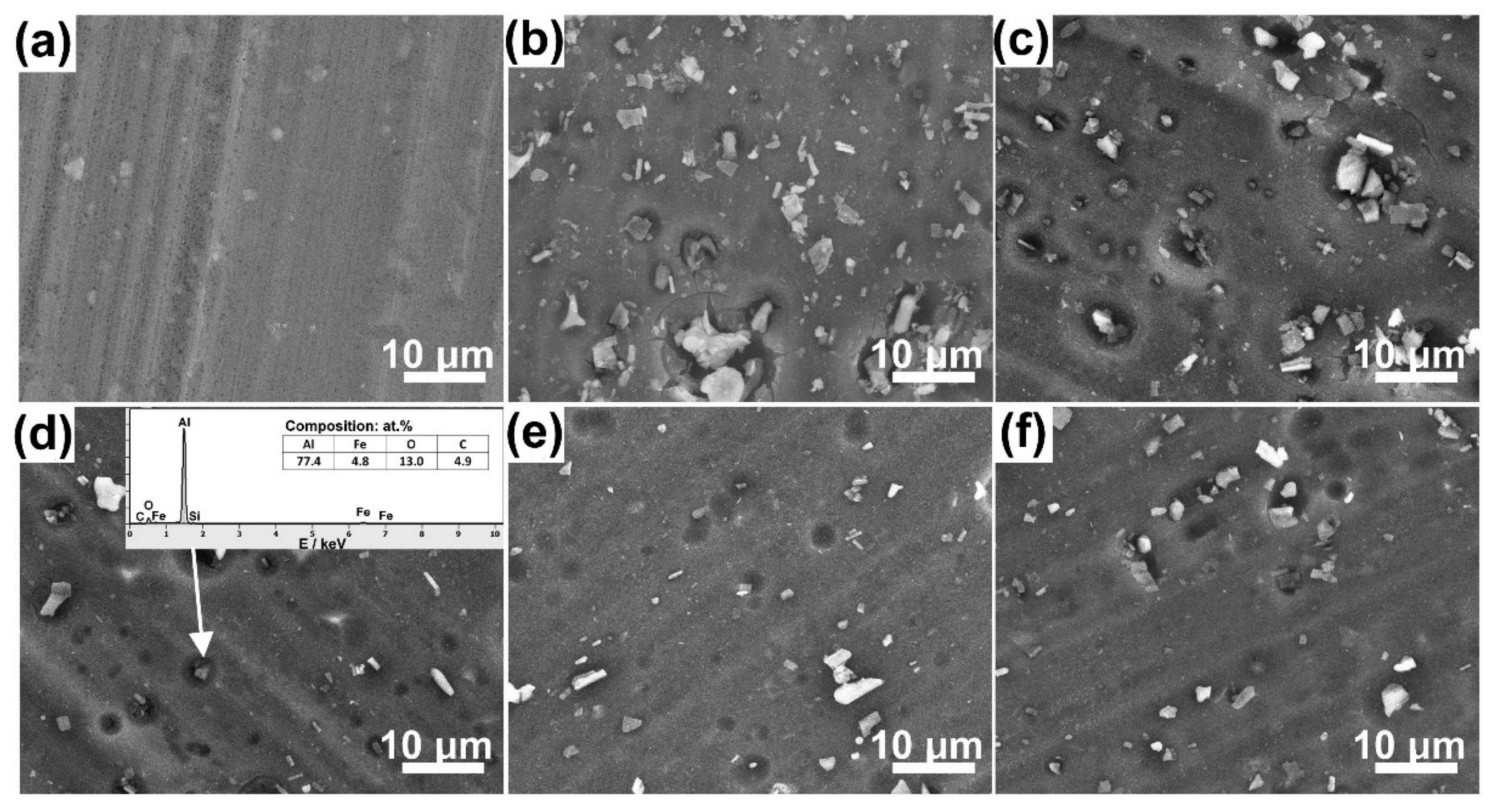
SEM micrographs illustrating the topography of the studied Al alloy samples: (**a**) as-polished, (**b**) corroded in 1 M NaOH, (**c**–**f**) corroded in 1 M NaOH with one of the studied inhibitors ((**c**) (**3**), (**d**) (**4**), (**e**) (**5**), (**f**) (**6**)). The inset is the EDX chemical map showing the chemistry of the intermetallic particles.

Next, SEM/EDX analyses were undertaken in order to define the changes in surface chemistry and topography resulting from exposure to corrosive media containing one of the studied corrosion inhibitors. The representative SEM micrographs are depicted in Figure 3. First, the SEM of the as-polished Al alloy sample is shown in Figure 3a, where the light spots represent the iron-rich intermetallic phase, which is cathodic to the aluminum matrix.

The general corrosion of aluminum alloys initially occurs at the galvanic microcells between the alloy matrix and the intermetallic phases. Thus, the iron-rich cathodic intermetallic phases appear as oxygen depolarization centers, with the aluminum alloy’s dissolution taking place around and in the direct vicinity of the cathodic particles. This process is illustrated in Figure 3b, which shows a micrograph taken for the corroded sample in absence of an inhibitor. There are no significant changes in topography outside of these galvanic microcells, due to the solubility of the corrosion products. Furthermore, based on the visual analysis, it should be noted that inhibitor (**3**) only partially restricts the corrosion process. The three remaining inhibitors—(**4**), (**5**) and (**6**)—provide a similar level of protection based on surface topography and the intensity of the galvanic microcell action.

Moreover, the energy-dispersive X-ray spectroscopy (EDX) studies, carried out to investigate the surface chemistry of each exposed sample, confirmed the highest share of aluminum and the lowest share of oxygen on the surface of the samples exposed to inhibitors (**4**) and (**6**), followed by the sample exposed to inhibitor (**5**). In particular, the alloy protected by inhibitor (**6**) featured a surface chemical composition very similar to that of the polished sample. The detailed information is given in Table 1 (the analyses were carried out on a surface area of approximately 3 mm^2^). The share of oxygen species was naturally the highest on the surface of Al samples exposed to corrosive media in the absence of any inhibitor. This might be due to the formation of thicker oxide films or the effect of surface roughness. The EDX analyses also confirmed the formation of galvanic microcells, with a high share of iron present in the intermetallic phases, as seen by the analysis in the inset of Figure 3d. Here, the share of iron may even exceed 5 at.%, while it should be noted that a share of the analyzed signal may originate from beneath the intermetallic phase, due to the depth of EDX analysis (up to 10 µm). This value is on average five times higher than the iron distribution on the surface of the studied alloys (see Table 1).

**Figure 4:**
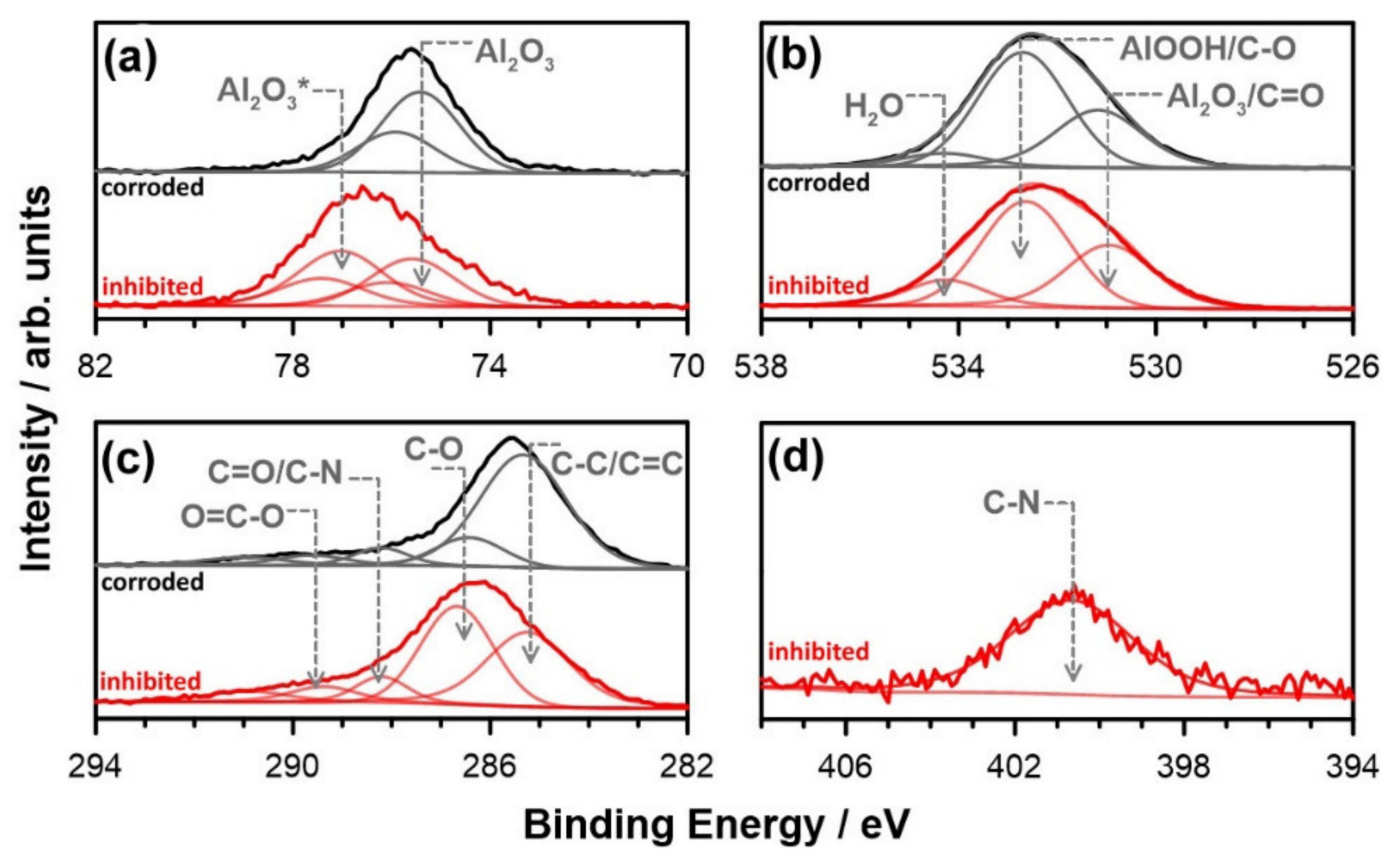
High-resolution XPS examination: (**a**) Al 2p, (**b**) O 1s, (**c**) C 1s and (**d**) N 1s of the Al alloy samples corroded in 1 M NaOH, with and without the addition of corrosion inhibitor (**4**).

Figure 4 presents the XPS plots of the Al alloy sample protected by corrosion inhibitor (**4**), compared with a freely corroded sample, in 1 M NaOH solution.

The Al 2p spectrum of the freely corroded Al sample in 1.0 M NaOH solution revealed the presence of a single chemical state, with Al 2p_3/2_ peaking at 75.4 eV—a value typical of native-grown Al_2_O_3_ oxides (Figure 4a). Since the corrosion studies were carried out in alkaline media, where the passive layer on the Al metal dissolved due to being thermodynamically unstable, the obtained XPS results indicate that no Al corrosion products were adsorbed on the surface of the studied samples. On the contrary, addition of the inhibitor compound led to the appearance of a second type of species, with Al 2p_3/2_ peaking at 77.2 eV. This component is often observed in alkaline and neutral electrolytes containing carboxyl species, proving the interaction of the inhibitor compound with the Al metal surface, and the formation of non-stoichiometric aluminum oxides [41,42,43,44]. Notably, the share of these Al_2_O_3_* species was over twice as large as the share of the native-grown oxide layer on the surface of the sample protected by inhibitor (**4**). The deconvoluted data are summarized in Table 2.

Interestingly, the oxygen chemistry of both protected and unprotected metal surfaces appeared very similar (see Figure 4b). The O 1s spectra were deconvoluted into three different components, hinting at multiple oxygen chemical states present on the electrode surface. The primary component at 532.8 eV lies in the energy range characteristic of hydrated aluminum oxides, but also of various compounds containing C-O bonding. Moreover, the second notable component, negatively shifted by −1.4 eV vs. the primary one, presents the superimposition of the signals originating from oxygen in Al_2_O_3_ and C=O bonded oxygen, typical of samples corroded in naturally aerated electrolytes. Finally, the small signal present at 534.4 eV is characteristic of chemically adsorbed water molecules. Its share is comparable, but slightly higher in the case of inhibitor-protected Al alloys.

The studied inhibitor molecules’ adsorption on the surface of the studied aluminum alloy is evident when comparing the N 1s and C 1s XPS spectra for both samples (Figure 4c,d). For the unprotected sample, the carbon content originates from the secondary corrosion processes between the Al dissolution products in alkaline media [Al(OH)_4_]^−^ and dissolved CO_2_, leading to the formation of carbonates, as well as adventitious carbon contamination in the air atmosphere. Thus, the comparative analysis is of primary importance. The intensity of the peaks originating from various C-O, C-N, and C=O bonds, which are present in the inhibitor molecule, was twice as high in the case of the Al alloy after exposure to the inhibitor-protected electrolyte [42,45,46]. Moreover, the high-resolution spectra recorded in the N 1s range allowed us to observe a spectral component in the binding energy range typical of C-N interaction, as in the case of the studied inhibitor molecules [47,48].

### 3.4. Computational Study

#### 3.4.1. DFT Modelling

**Figure 5:**
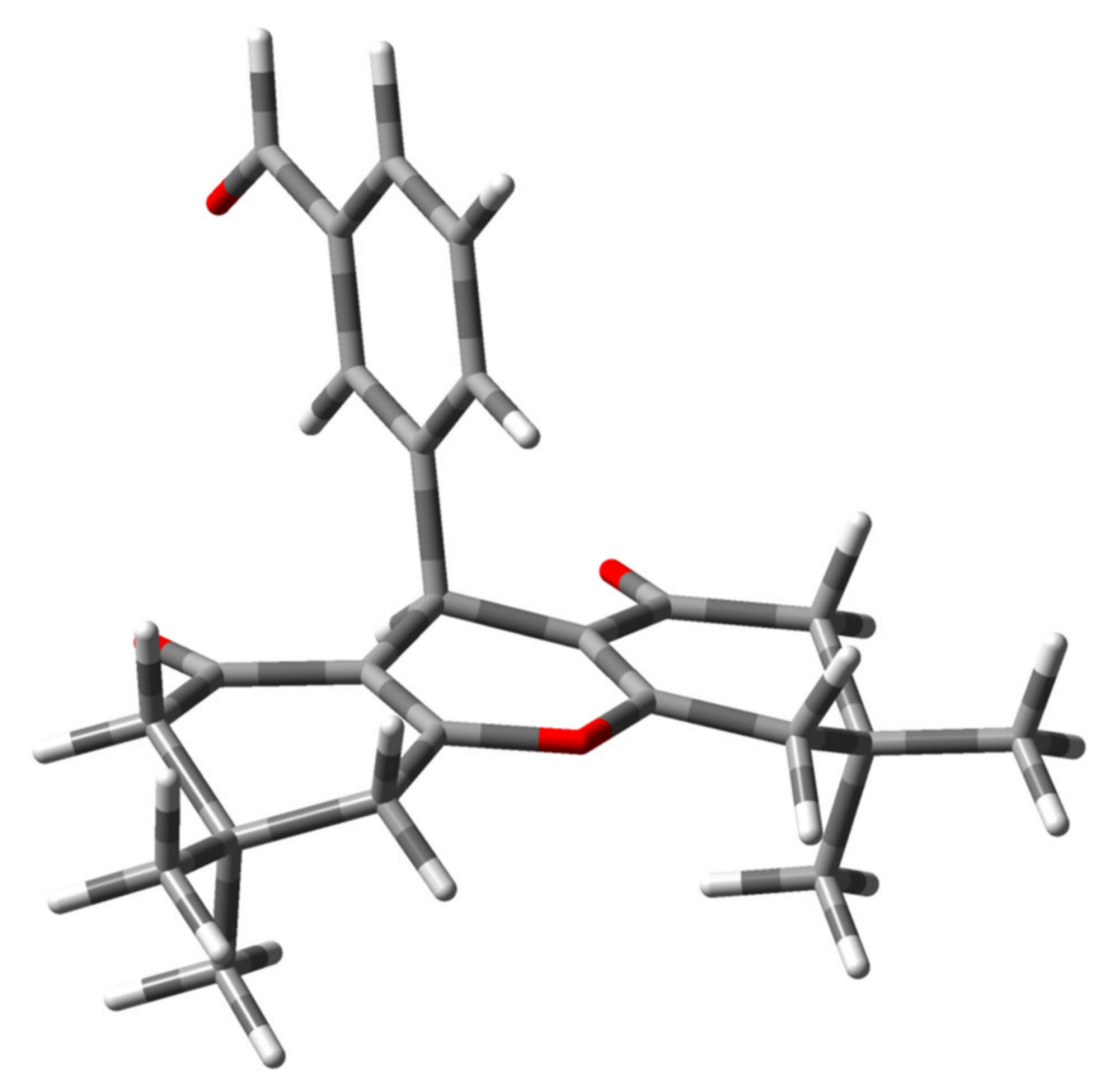
The optimized geometry of compound (**3**), as a representative example.

**Figure 6:**
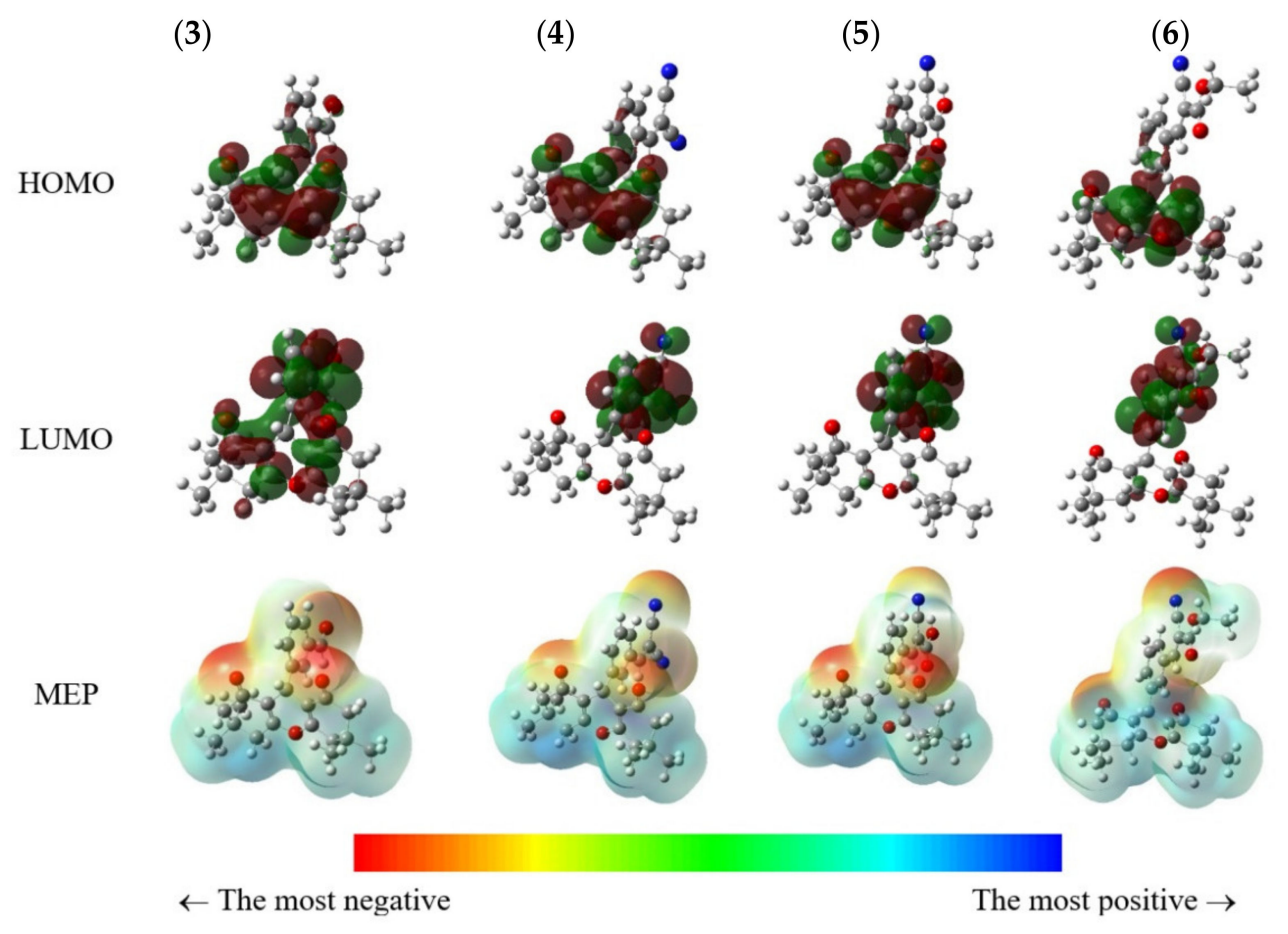
The calculated FMOs and MEP of the investigated xanthenes using B3LYP/6-311++G(2d,2p) in the aqueous phase.

Quantum chemical calculations were used to determine some elementary properties of isolated organic molecules in the aqueous phase before interacting with a metal surface, as illustrated in Table 3 and Figure 5 and Figure 6. According to the optimized geometry of all studied xanthenes, the benzylidene ring was observed to be at a right angle with respect to the 9*H*-xanthene cleft (Figure 5). This non-planarity may lead to a lack of complete correlation between the descriptors and the efficiency of experimental corrosion inhibition since, in this case, it cannot be guaranteed that all heteroatoms in a particular compound are not in direct contact with the metallic surface.

The electron density distribution of frontier molecular orbitals (FMOs) and molecular electrostatic potential are depicted in Figure 6. The involvement of methylene malononitrile fragments in the structures compounds of (**4**)–(**6**) cause these molecules to belong to donor–acceptor (D–A) systems. For this reason, one can expect the HOMO to be localized on the donor moiety (xanthene), whereas the LUMO would be situated on the acceptor moiety (methylene malononitrile). The D–A features of these molecules support the idea of their ability to donate and back-donate charges to/from the metallic surface.

Molecular electrostatic potential (MEP) illustrates the 3D charge distributions of molecules. These maps allow us to visualize variably charged regions of a molecule. Knowledge of the charge distributions can be used to determine how molecules interact with the metallic surface—that is, to predict electrophilic attack from the metallic side. The ESP surfaces of compounds (**3**)–(**6**) in the aqueous phase are shown in Figure 6. The blue color in the ESP plots represents the maximum amount of the positive region where the nucleophilic reaction takes place, while the reddish region represents the negative region where the electrophilic reaction occurs. It is clear that oxygen atoms carry the highest electron density in all cases; thus, it is expected that these atoms will participate vigorously in the adsorption process on the Al surface.

On the other hand, the value of *E*_HOMO_ energy indicates the electron-donating ability of the inhibitor molecules. The present case obeyed the order (**4**) > (**5**) > (**3**) > (**6**), in partial agreement with the experimental observations. Unlike *E*_HOMO_, the *E*_LUMO_ value is a measure of the electron affinity of the inhibitor molecule. It was observed that *E*_LUMO_ showed the opposite trend to *E*_HOMO_. Moreover, ΔE is another critical parameter that describes the interaction between the inhibitor molecule and the metallic surface. In general, an inhibitor with a lower ΔE value is associated with high chemical reactivity and, therefore, with better inhibition performance compared to an inhibitor with a higher ΔE value. The ΔE of the investigated molecules was subject to the same order of inhibition effect. Conversely, the dipole moment (DM) is not a straightforward parameter in controlling the inhibition efficiency [49].

**Figure 7:**
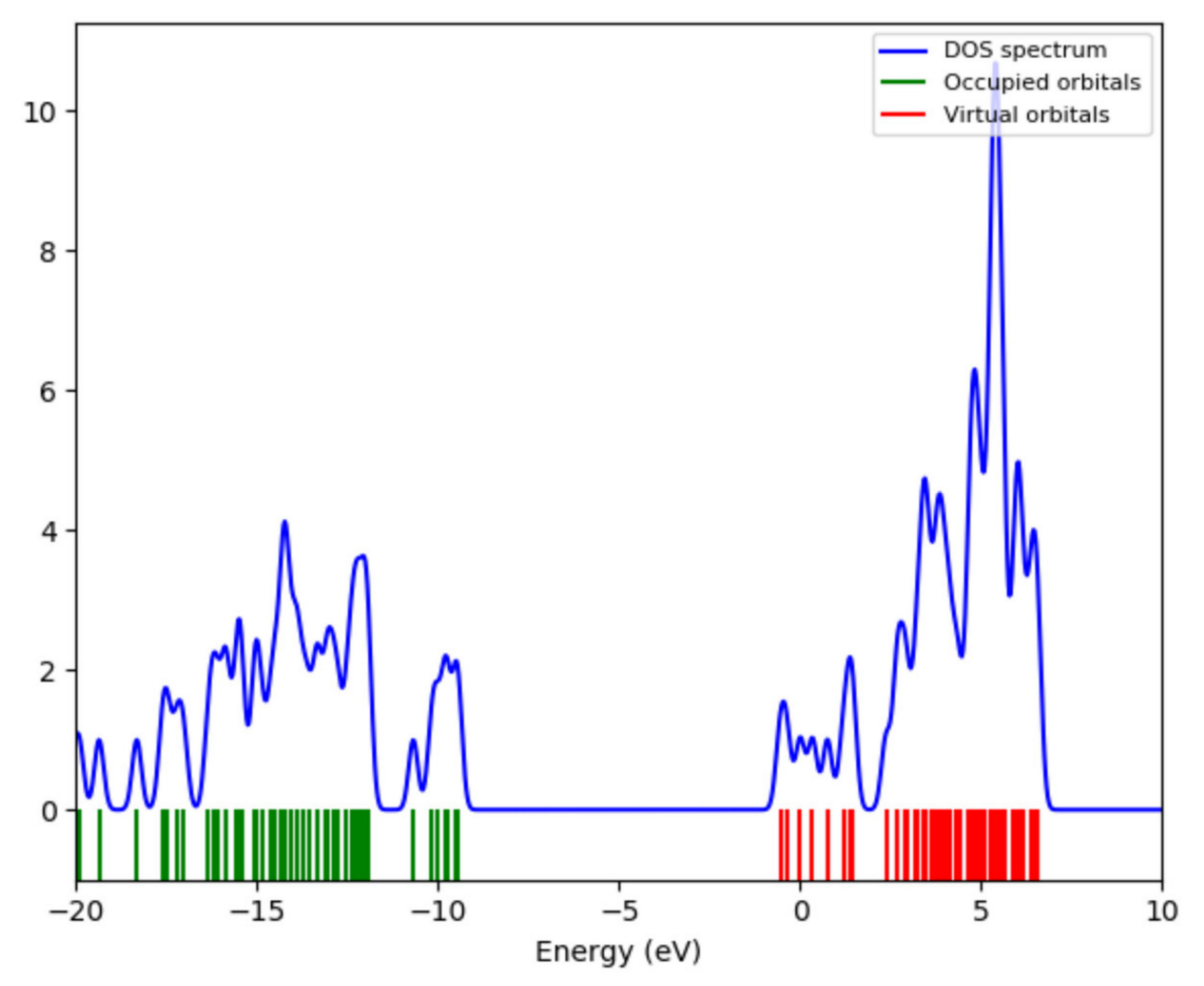
The density of states (DOS) spectrum of compound (**3**), as a representative example.

It is common for the electronic structure of aromatic molecules to contain quasi-degenerate energy levels of HOMO and LUMO. Thus, the HOMO and LUMO may not lead to a realistic description of the frontier orbitals in the boundary region. For this reason, the density of states (DOS) was calculated based on B3LYP/6-311G(d,p) to describe this phenomenon using the Gauss Sum 3.0 software [50]. Figure 7 shows the DOS spectrum of compound (**3**) as a representative example, while the DOS spectra of the other compounds can be found in the Supplementary Materials. There are many DOS spectrum peaks, meaning that the orbital molecular energy levels are essentially similar. Therefore, the studied xanthene compounds easily interact with the Al surface.

The molecular volume (M_V_) indicates the potential for covering the surface of the metal with the inhibitor. A large M_V_ translates into higher protection efficiency due to the increased surface coverage [51]. Accordingly, the computed M_V_ follows the same order as the experimental inhibition efficiency.

#### 3.4.2. Monte Carlo Simulations

The adsorption process is the success of the molecule in paralleling its structure as closely as possible to the metal surface to reduce the area of contact of the corrosion elements (e.g., H_2_O, acidic or alkaline medium) with the surface. Thus, we aimed to distinguish between the capacities of the studied inhibitors to adsorb to the Al (111) surface by performing Metropolis Monte Carlo simulations and comparing them with relevant practical results. Table 4 shows the adsorption descriptors of the studied compounds, including total, adsorption, rigid adsorption, and deformation energies. Adsorption energy is the most relevant energy parameter for adsorption, defined as the sum of the rigid adsorption energy before and after the relaxation of the adsorbates on the surface. As shown in Table 4_,_ the adsorption of compounds (**3**)–(**6**) on the Al surface was spontaneous, as reflected in the negative adsorption energy. The highly negative adsorption energy indicates that the interactions between the studied adsorbates and the Al surface were strong. By taking the surface energy of Al as zero, the differential energy (dE_ad_/dN_i_) can be defined as the energy of substrate–adsorbate configuration when one adsorbate is removed.

Comparing the average adsorption energy of the adsorbates (~−714 kcal/mol) with the differential desorption energy (~−90 kcal/mol) indicates that the adsorption process is highly favored and irreversible. Accordingly, the distinction between the dE_ad_/dN_i_ of inhibitors is pointless. Although corrosive elements such as H_2_O and NaOH are present in the medium, the inhibitors adsorb preferably on the Al (111) surface without significant competition, because the adsorption requires much less energy. Regarding the deformation adsorption energy, compound (**4**) has a higher degree of deformation than the other compounds, attributed to its higher dipole moment value (Table 3). On the other hand, the adsorption energy of compound (**4**) is the lowest, at around −739 kcal/mol, compared to −720 kcal/mol for (**5**), −705 kcal/mol for (**6**), and −690 kcal/mol for (**3**). This indicates that compound (**4**) requires less energy for adhesion onto the Al (111) surface compared to the other inhibitors, which require more energy (E_ads_) for adsorption. We can conclude that the effectiveness of the xanthene inhibitors follows the order (**4**) > (**5**) > (**6**) > (**3**), which matches with the experimental findings.

**Figure 8:**
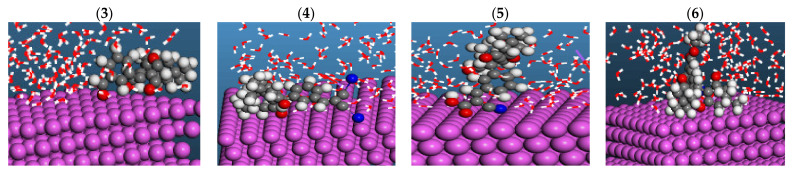
The most stable configurations for the adsorption of xanthene molecules on Al (111) surfaces in alkaline media.

To explain this order, let us inspect the side and top views of the adsorption configuration (Figure 8). Because the investigated xanthenes have non-planar geometries, it is rational to conclude that not all hetero atoms will contact the Al (111) surface. Compounds (**4**)–(**6**) attach to the surface via only two heteroatoms, namely, a nitrile nitrogen atom and an etheric oxygen/ketonic oxygen atom. Moreover, the phenylene moiety tends to be coplanar with the surface. The superiority of the adsorption of compound (**4**) over compounds (**5**) and (**6**) can be attributed to the participation of ketone oxygen in the adsorption process of the former, as opposed to the etheric oxygen in the other compounds. This can be explained based on the fact that a carbonyl group can interact with both direct donations and back-donations with the metallic surface, while etheric oxygen uses only the direct donation property. On the other hand, compound (**3**) has the lowest adsorption interaction because it does not involve the nitrile group. It is known that the nitrogen atom has a higher donating power than the oxygen atom.

## 4. Conclusions

A new benzaldehyde xanthene derivative 3-(3,3,6,6-tetramethyl-1,8-dioxo-2,3,4,5,6,7,8,9-octahydro-1*H*-xanthen-9-yl)benzaldehyde, labelled compound (**3**), was synthesized using a diprotic Brønsted-acid-catalyzed reaction of dimedone and isophthaldehyde. Basic condensation reaction of compound (**3**) with active nitrile function allowed the preparation of novel cyano-benzylidene xanthene clusters (**4**)–(**6**). The promising entities were fully characterized for structural elucidation. The inhibitory action of compounds (**3**), (**4**), (**5**), and (**6**) against the uniform corrosion of aluminum in an alkaline medium (1.0 M NaOH) was electrochemically evaluated utilizing the methods of Tafel extrapolation and linear polarization resistance (LPR). The efficiency of each tested inhibitor against the alkaline uniform corrosion of Al increased upon increasing its concentration. Maximum inhibition efficiency values of 91.8, 94.9, 96.8, and 98.9% were achieved for (**3**), (**5**), (**6**), and (**4**), respectively, at an inhibitor concentration of 2.0 mM. The examined xanthenes were classified as mixed-type inhibitors that predominantly control the anodic process, based on the results of polarization experiments. XPS analysis confirmed the inhibitor compounds’ adsorption and the subsequent formation of a protective film of inhibitor molecules. The topography of the corroded and inhibited Al surfaces revealed that in the presence of inhibitor (**4**)—the best performing inhibitor—the corroded patches on the electrode surface were effectively decreased.

On the other hand, the computational findings indicate that due to the non-planarity of xanthene’s geometry, only two heteroatoms plus the phenylene ring participate in the adsorption process. Monte Carlo simulations showed that the adsorption energy of the studied xanthenes followed the order (**4**) > (**5**) > (**6**) > (**3**), which is consistent with the experimental results. This highlights the importance of the presence of a nitrile group in the structure of aluminum inhibitors to improve their performance. Furthermore, the multipeak DOS spectra of the investigated inhibitors indicate that not only FMO, but also the proximal orbitals are available for interaction with the metallic surface. 

## Figures and Tables

**Table 1:** The EDX chemical composition (in at.%) of the surface of Al samples polished and exposed to the corrosive media in the absence or presence of the studied corrosion inhibitors.

	Al (at.%)	Fe (at.%)	O (at.%)	C (at.%)
Polished	96.0	0.9	3.1	0.0
Corroded	78.8	0.9	18.3	2.0
(**3**)	78.6	1.2	12.5	7.8
(**4**)	86.7	1.0	9.0	3.4
(**5**)	85.5	1.1	10.3	3.1
(**6**)	91.5	1.0	5.3	2.1

**Table 2:** Surface chemical composition (in at.%) based on the XPS analysis of the Al alloy samples corroded in 1 M NaOH with and without the addition of inhibitor (**5**).

	C 1s			Al 2p_3/2_		O 1s			N 1s
	C-C /C=C	C-O	C-N /C=O	Al_2_O_3_ /AlOOH	Al_2_O_3_ *	Al_2_O_3_ /C=O	AlOOH/CO	H_2_O_ads_	C-N
BE/eV	285.4	286.8	288–291	75.4	77.2	531.4	532.8	534.4	400.5
Corroded	24.0	7.3	4.0	21.9	-	13.9	24.8	3.1	-
(**4**)	14.2	14.5	7.8	8.5	20.5	14.1	23.2	4.5	2.2

**Table 3:** The B3LYP/6-311++G(2d,2p) quantum chemical descriptors of the investigated xanthenes in the aqueous phase.

Molecule	E_HOMO_, eV	E_LUMO_, eV	∆E, eV	DM, D
(**3**)	−6.71	−2.13	4.58	11.12
(**4**)	−6.74	−2.97	3.78	13.55
(**5**)	−6.74	−2.89	3.85	10.09
(**6**)	−6.70	−2.74	3.96	12.55

**Table 4:** Monte Carlo descriptors for Al (111)/inhibitor/(1 NaOH/555 H_2_O) systems. All values are in kcal/mol.

Inhibitor	Total Energy (kcal/mol)	Adsorption Energy (kcal/mol)	Rigid Adsorption Energy (kcal/mol)	Deformation Energy (kcal/mol)	dE_ad_/*d*N_i_		
					Inhibitor	NaOH	H_2_O
(**3**)	−597	−690	−680	−10	−78	−35	−11
(**4**)	−645	−739	−734	−5	−98	−32	−13
(**5**)	−623	−720	−713	−4	−95	−39	−13
(**6**)	−612	−705	−697	−8	−89	−33	−12

## Data Availability

All data are available on request.

## Supplementary Materials

The following are available online at https://www.mdpi.com/article/10.3390/molecules27175733/s1, S1: General procedure of the synthesis of the studied compounds, S2: ^1^H-NMR and FTIR spectra of the studied xanthene derivatives, S3: Electrochemical kinetic parameters based on polarization studies, S4: Cartesian coordinates for the optimized geometry of the studied xanthene.

## Sample Availability

Samples of the compound are available from the authors on demand.
