# Supplementary material for: Synthesis of Cyano-Benzylidene Xanthene Synthons Using a Diprotic Brønsted Acid Catalyst, and Their Application as Efficient Inhibitors of Aluminum Corrosion in Alkaline Solutions
Source: Molecules. 2022 Sep 5;27(17):5733. doi: 10.3390/molecules27175733 (PMC9457813; doi:10.3390/molecules27175733)
Supplement: Supplementary file 1 [file molecules-27-05733-s001.zip › molecules-1654816-supplementary.pdf]

Article

Synthesis of Cyano-Benzylidene Xanthene Synthons Using a Diprotic Brønsted Acid Catalyst, and Their Application as Efficient Inhibitors of Aluminum Corrosion in Alkaline Solutions

Mohammed A. Amin [1,*], Gaber A. M. Mersal [1], Morad M. El-Hendawy [2], Abdallah A. Shaltout [3], Ali Badawi [3], Johan Boman [4], Adil A. Gobouri [1], Murat Saracoglu [5], Fatma Kandemirli [6], Rabah Boukherroub [7], Jacek Ryl [8,*] and Mohamed E. Khalifa [1]

1 Department of Chemistry, College of Science, Taif University, P.O. Box 11099, Taif 21944, Saudi Arabia
2 Chemistry Department, Faculty of Science, New Valley University, Kharga 72511, Egypt
3 Department of Physics, College of Science, Taif University, P.O. Box 11099, Taif 21944, Saudi Arabia
4 Department of Chemistry and Molecular Biology, Atmospheric Science, University of Gothenburg, 412 96 Gothenburg, Sweden
5 Faculty of Education, Erciyes University, 38039 Kayseri, Turkey
6 Department of Biomedical Engineering, Faculty of Engineering and Architecture, Kastamonu University, 37150 Kastamonu, Turkey
7 University of Lille, CNRS, Centrale Lille, University Polytechnique Hauts-de-France, UMR 8520-IEMN,
F-59000 Lille, France
8 Division of Electrochemistry and Surface Physical Chemistry, Institute of Nanotechnology and Materials Engineering, Gdansk University of Technology, Narutowicza 11/12, 80-233 Gdansk, Poland
* Correspondence: mohamed@tu.edu.sa (M.A.A.); jacek.ryl@pg.edu.pl (J.R.)

S1. General procedure of the synthesis of the studied compounds

S1.1. Synthesis of 3-(3,3,6,6-tetramethyl-1,8-dioxo-2,3,4,5,6,7,8,9-octahydro-1H-xanthen-9-yl)benzaldehyde (**3**)

The synthesis of compound **3** was performed by the reaction of 5,5-dimethylcyclohexan-1,3-dione (**1**; 20 mmol, 2.80 g) with isophthaldehyde (**2**; 10 mmol, 1.34 g) and oxalic acid solution as a catalyst (10 mmol, 0.99 g) at 80 °C in a water bath under constant stirring for 40 min. The reaction mixture was then poured onto ice-cold water. The solid product was collected, filtered off, dried and recrystallized from ethanol as buff crystals in 67% yield; M.p. 156-158 °C; ^{1}H-NMR (DMSO-d_6) (δ/ppm): 2.36 (s, 12H, 3Me), 2.49 (s, 4H, Xanthene$_{(C4,5)}$-H), 3.35 (s, 4H, Xanthene$_{(C2,7)}$-H), 7.72-7.91(m, 4H, Ar-H), 9.88 (s, 1H, CHO). ^{13}C-NMR (300 MHz,): 27.5 (4 Me), 32.2 (C-3,6-xanth), 38.7, 51.6 (4CH_2), 39.1 (C-9-xanth), 113.8, 126.8, 129.1, 130.1, 133.4, 139.3, 142.5, 155.1 (5C=C), 191.1 (CHO), 198.8 (2C=O); MS (*m/z*, %): 379.19 (M+1, 26.4). Anal. Calcd. for $C_{24}H_{26}O_4$ (378.46) C, 76.17; H, 6.92; O, 16.91%. Found: C, 76.01; H, 6.75; O, 16.81%.

S1.2. General procedure for the synthesis of 2-(4-(12H-dibenzo[c]xanthen-7-yl)benzylidene) derivatives (compounds 4-6).

The xanthene derivative (**3**; 10 mmol, 3.86 g) was allowed to react with a molar ratio of the active nitrile compounds, malononitrile (0.66 g), cyanoacetic acid (0.85 g) or ethylcyanoacetate (1.13 g). The reaction mixture was kept under magnetic stirring at ambient temperature for 2 h in 30 mL ethanol and piperidine (2 mL) as basic catalyst. The mixture was poured onto ice-cold water and then acidified with dilute hydrochloric acid (2 M). The product was filtered off, dried and recrystallized from ethanol, yielding the cyanobenzylidene xanthenes (compounds **4-6)**, respectively.

2-(3-(3,3,6,6-Tetramethyl-1,8-dioxo-2,3,4,5,6,7,8,9-octahydro-1H-xanthen-9-yl)benzylidene)malononitrile (**4**). Faint yellow crystals; Yield: 73% (3.10 g); M.p. >250 °C. IR: ν_{max}=2871(=CH Ar), 2190, (C≡N$_{str.}$), 1666 (C=O$_{str.}$ aldehydic), 1476 (CH_2), 1365 (CH_3) and 1213 (C-O xanthene ring) cm^{-1}. ^{1}H-NMR (300 MHz, DMSO-d_6) (δ/ppm): 2.38 (s, 12H, 4 Me); 2.51 (s, 4H, Xanthene$_{(C4,5)}$-H), 3.37 (s, 4H, Xanthene$_{(C2,7)}$-H); 7.35-7.84 (m, 4H, Ar-H); 7.93 (s, 1H, olefinic proton). ^{13}C-NMR (75 MHz) δ: 199.12, 159.11, 147.12, 136.13, 128.10, 124.95, 108.31. MS (*m/z*, %): 427.20 (M+1, 29.6). Anal. calc. for $C_{27}H_{25}N_2O_3$ (426.51) C, 76.03; H, 6.14; N, 6.57 (Found: C, 76.45; H, 6.56; N, 6.87).

2-Cyano-3-(3-(3,3,6,6-tetramethyl-1,8-dioxo-2,3,4,5,6,7,8,9-octahydro-1H-xanthen-9-yl)phenyl)acrylic acid (5). Faint yellow crystals; Yield: 68% (3.02 g); M.p. 120-121 °C. IR: ν_{max}=3392 ($OH_{str.}$), 2226, ($C{\equiv}N_{str.}$), 1666 ($C{=}O_{str.}$carboxylic), 1470 (CH_2), 1365 (CH_3) and 1213 (C-O xanthene ring) cm^{-1}. ^{1}H-NMR (300 MHz, DMSO-*d*$_6$) (δ/ppm): 2.38 (s, 12H, 4 CH_3); 2.51 (s, 4H, Xanthene$_{(C4,5)}$-H), 3.35 (s, 4H, Xanthene$_{(C2,7)}$-H); 7.74-7.95 (m, 4H, Ar-H); 8.53 (s, 1H, olefinic proton); 12.45 (s, 1H, COOH). ^{13}C-NMR (75 MHz) δ: 199.12, 159.11, 147.12, 136.13, 128.10, 124.95, 108.31. MS (*m/z*, %): 445.19 (M^+, 4.31). Anal. calc. for $C_{27}H_{27}NO_5$ (445.51) C, 72.79; H, 6.11; N, 3.14 (Found: C, 72.65; H, 6.33; N, 3.46).

Ethyl-2-cyano-3-(3-(3,3,6,6-tetramethyl-1,8-dioxo-2,3,4,5,6,7,8,9-octahydro-1H-xanthen-9-yl)phenyl)acrylate (6). Yellow crystals; Yield: 81% (4.73 g); M.p. 134-136 °C; IR: ν_{max}=2957 (=CH Ar), 2232, ($C{\equiv}N_{str.}$), 1666 ($C{=}O_{str.}$ aldehydic), 1447 (CH_2), 1365 (CH_3) and 1213 (C-O xanthene ring) cm^{-1}. ^{1}H-NMR (300 MHz, DMSO-*d*$_6$) (δ/ppm): 1.28 (s, 12H, 4 CH_3); 1.31 (t, 3H, CH_3); 2.51 (s, 4H, Xanthene$_{(C4,5)}$-H), 3.44 (s, 4H, Xanthene$_{(C2,7)}$-H); 4.25 (q, 2H, CH_2); 7.36-7.95 (m, 4H, Ar-H); 8.51 (s, 1H, olifinic proton). ^{13}C-NMR (75 MHz) δ: 199.12, 159.11, 147.12, 136.13, 128.10, 124.95, 108.31. MS (*m/z*, %): 474.22 (M+1, 31.9). Anal. calc. for $C_{29}H_{31}NO_5$ (473.56) C, 73.55; H, 6.60; N, 2.96 (Found: C, 73.45; H, 6.54; N, 3.26).

S2. ^{1}H-NMR and FTIR spectra of the studied xanthene derivatives

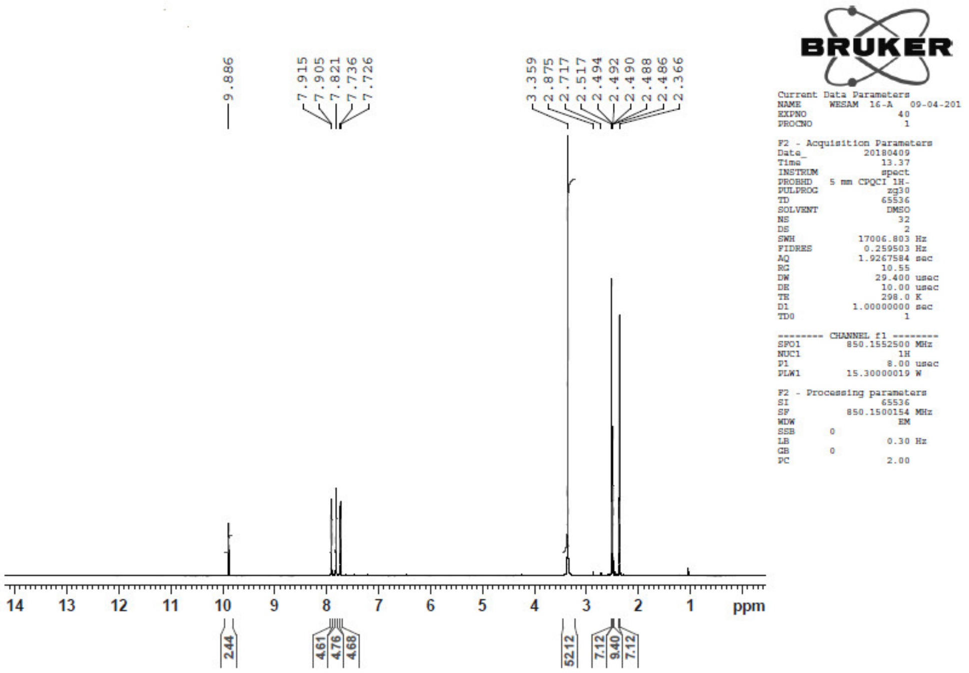

Figure S1. ^{1}H-NMR spectrum of benzaldehyde xanthene derivative **(3)**.

The xanthene-benzaldehyde **(3)** is hence incorporated in a set of reactions with different active nitriles, malononitrile, cyanoacetic acid or ethylcyanoacetate in presence of basic catalyst (piperidine**)**, to synthesize derivatives **(4-6)**, as shown in **Scheme 2**.

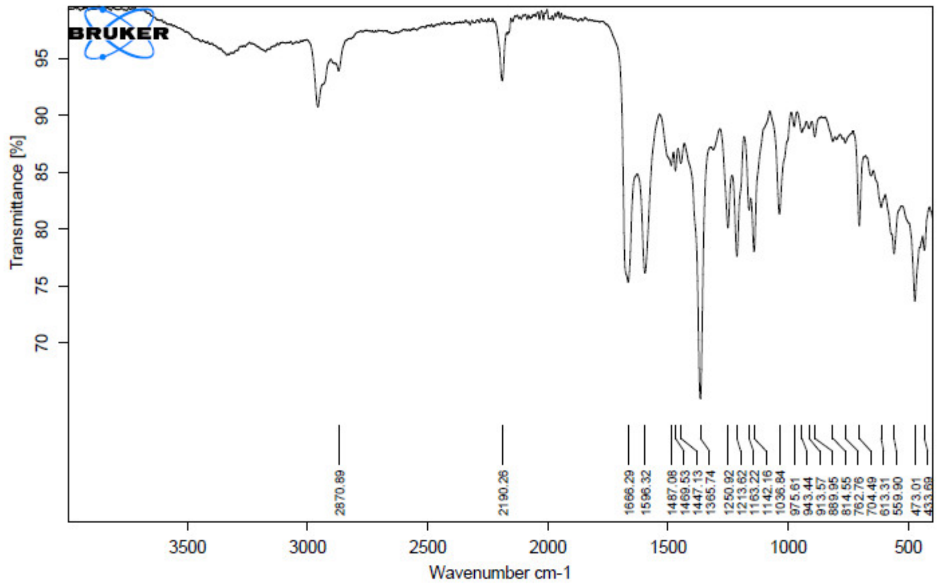

Figure S2. FTIR spectrum of malononitrile xanthene derivative **(4)**.

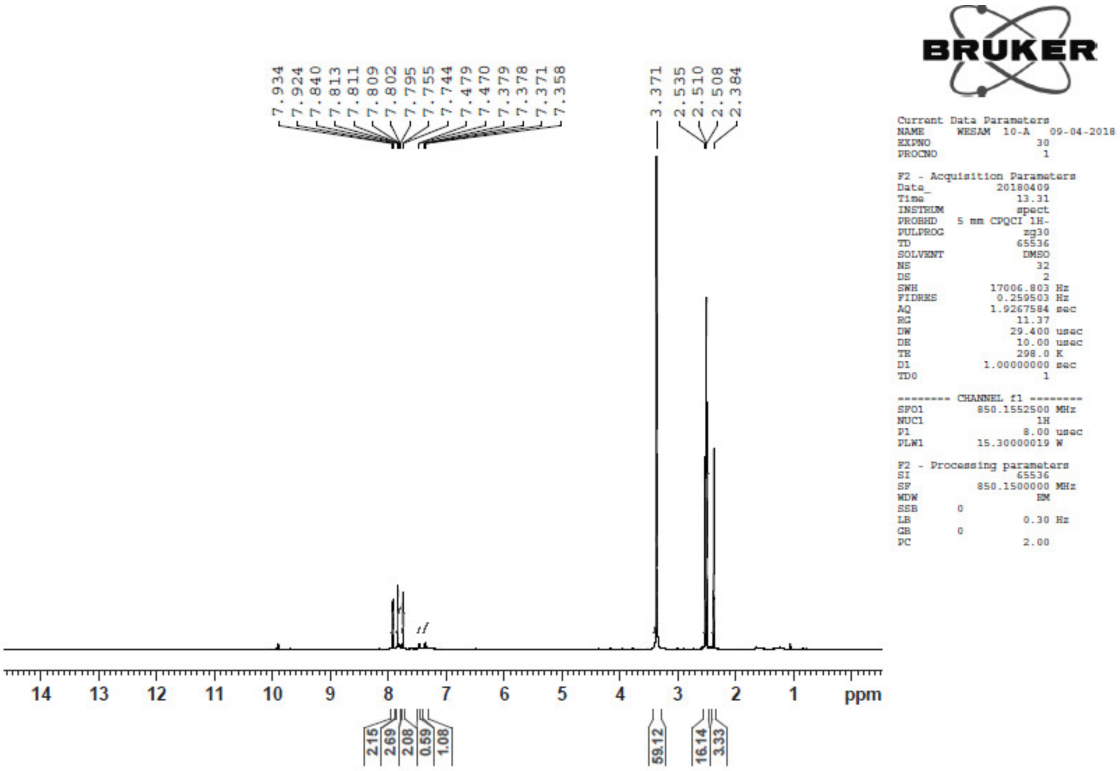

Figure S3. ^{1}H-NMR spectrum of malononitrile xanthene derivative **(4)**.

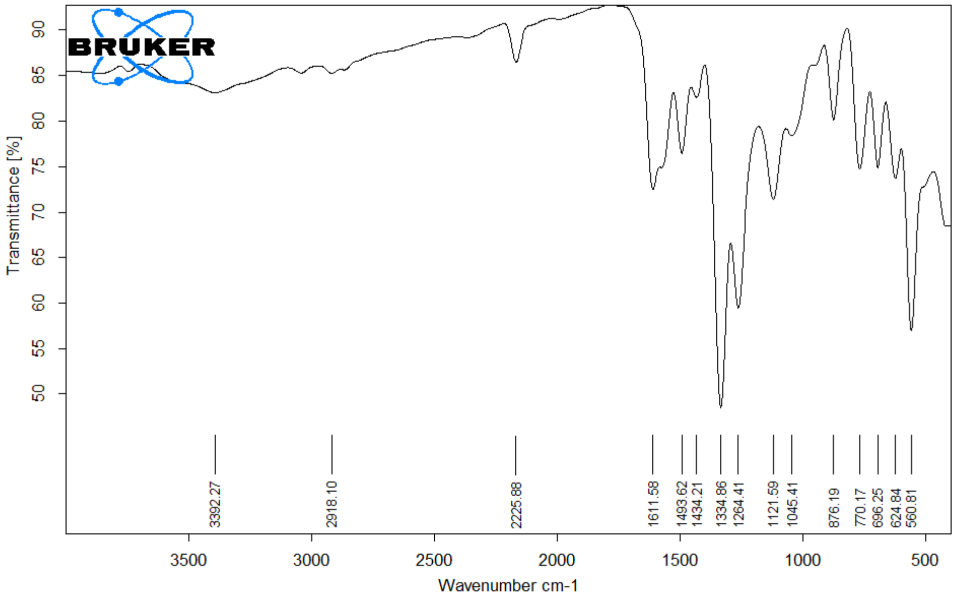

Figure S4. FTIR spectrum of acrylic acid xanthene derivative **(5)**.

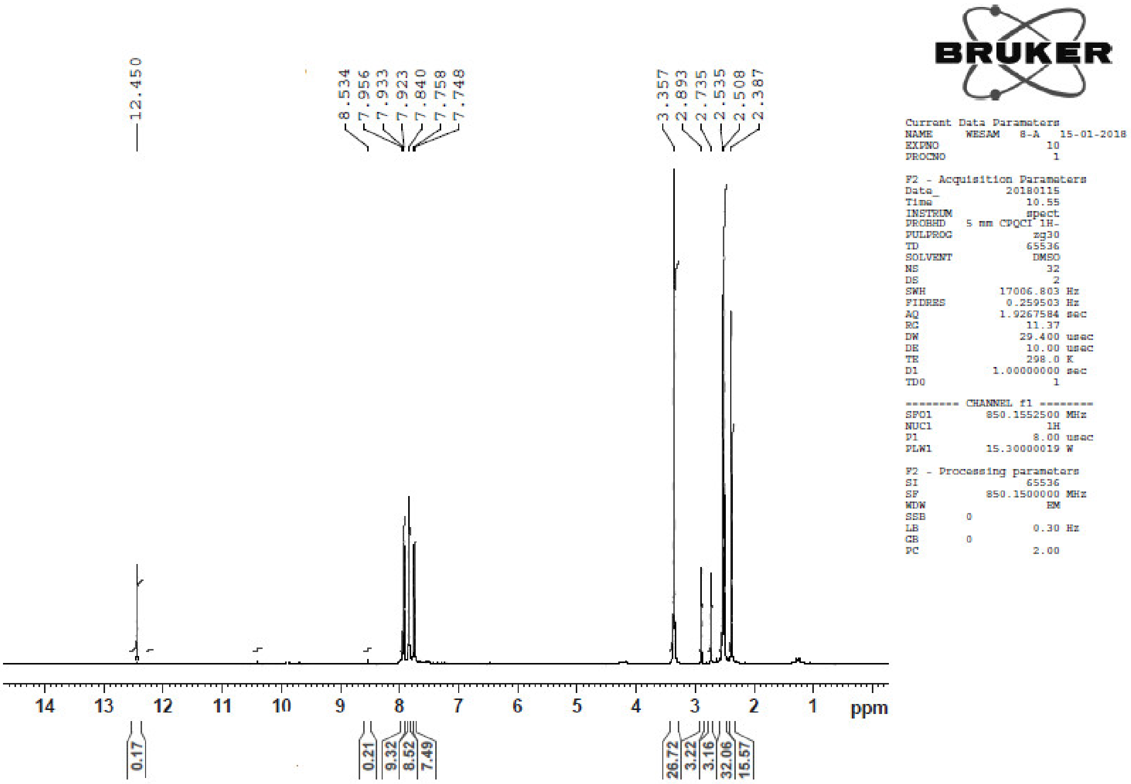

Figure S5. ^{1}H-NMR plot of acrylic acid xanthene derivative **(5)**.

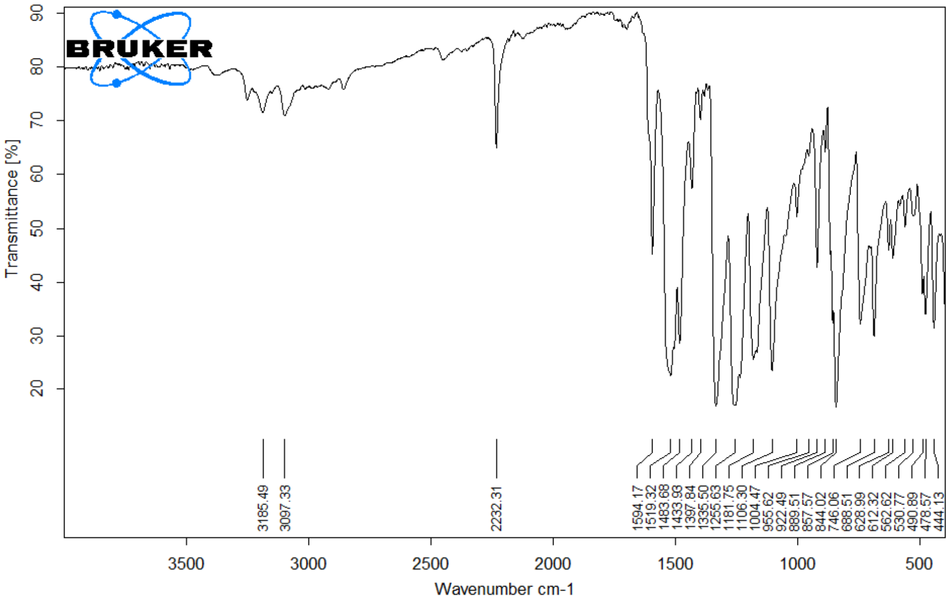

Figure S6. FTIR spectrum of acrylate xanthene derivative **(6)**.

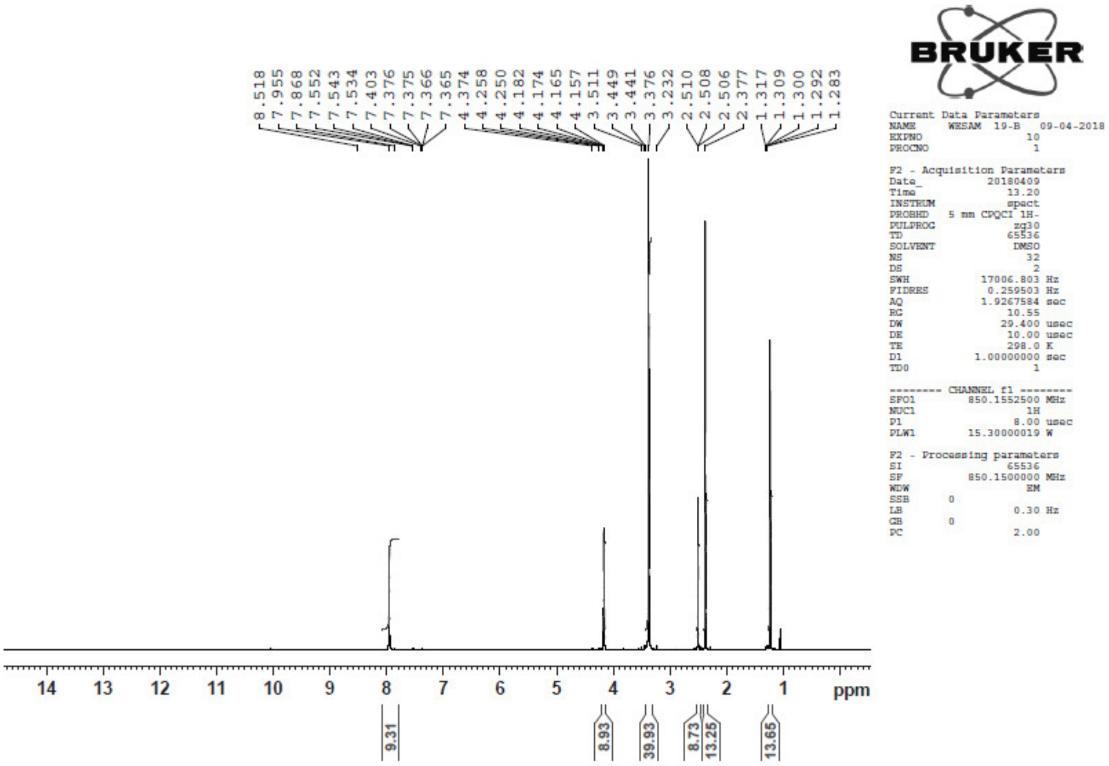

Figure S7. ^{1}H-NMR spectrum of acrylate xanthene derivative **(6)**.

S3. Electrochemical kinetic parameters based on polarization studies

Table S1 – Various electrochemical kinetic parameters recorded for compound **3**, derived from polarization measurements employing the Tafel extrapolation and LPR methods.

[Inhibitor] / mM	Tafel extrapolation method						LPR method			
	E_{corr} / V *vs.* Ag/AgCl	j_{corr} / A cm^{-2}	β_c / mV dec^{-1}	β_a / mV dec^{-1}	υ / mm yr^{-1}	*I*(%)	j_{corr} / A cm^{-2}	R_p / Ω cm^2	υ / mm yr^{-1}	*I*(%)
**0.0**	-1.520	1.26×10^{-3}	-0.249	0.241	13.73	----	1.17×10^{-3}	45.45	12.75	-----
**0.1**	-1.482	7.7×10^{-4}	-0.245	0.290	8.39	38.9	7.04×10^{-4}	81.91	7.67	39.8
**0.2**	-1.469	6.4×10^{-4}	-0.259	0.219	6.98	49.2	5.66×10^{-4}	91.03	6.17	51.6
**0.5**	-1.452	2.78×10^{-4}	-0.256	0.260	3.03	77.9	2.38×10^{-4}	235.34	2.59	79.7
**1.0**	-1.427	1.46×10^{-4}	-0.273	0.250	1.59	88.4	1.19×10^{-4}	476.17	1.30	89.8
**2.0**	-1.399	1.03×10^{-4}	-0.261	0.196	1.12	91.8	8.78×10^{-5}	553.6	0.96	92.5

Table S2 – Various electrochemical kinetic parameters recorded for compound **5**, derived from polarization measurements employing the Tafel extrapolation and LPR methods.

[Inhibitor] / mM	Tafel extrapolation method						LPR method			
	E_{corr} / V *vs.* Ag/AgCl	j_{corr} / A cm^{-2}	β_c / mV dec^{-1}	β_a / mV dec^{-1}	υ / mm yr^{-1}	*I*(%)	j_{corr} / A cm^{-2}	R_p / Ω cm^2	υ / mm yr^{-1}	*I*(%)
**0.0**	-1.520	1.26×10^{-3}	-0.249	0.241	13.72	----	1.17×10^{-3}	45.45	12.74	-----
**0.1**	-1.489	6.72×10^{-4}	-0.243	0.288	7.32	46.7	6.44×10^{-4}	88.86	7.015	44.9
**0.2**	-1.464	6.12×10^{-4}	-0.257	0.221	6.67	51.4	5.53×10^{-4}	93.3	6.02	52.7
**0.5**	-1.446	2.2×10^{-4}	-0.255	0.262	2.39	82.6	2.23×10^{-4}	251.6	2.43	81.0
**1.0**	-1.422	9.07×10^{-5}	-0.270	0.255	0.99	92.8	9.83×10^{-5}	579.3	1.07	91.6
**2.0**	-1.383	6.43×10^{-5}	-0.263	0.199	0.70	94.9	7.72×10^{-5}	637.2	0.84	93.4

Table S3 – Various electrochemical kinetic parameters recorded for compound **6**, derived from polarization measurements employing the Tafel extrapolation and LPR methods.

[Inhibitor] / mM	Tafel extrapolation method						LPR method			
	E_{corr} / V *vs.* Ag/AgCl	j_{corr} / A cm^{-2}	β_c / mV dec^{-1}	β_a / mV dec^{-1}	υ / mm yr^{-1}	*I*(%)	j_{corr} / A cm^{-2}	R_p / Ω cm^2	υ / mm yr^{-1}	*I*(%)

**0.0**	-1.520	1.26×10^{-3}	-0.249	0.241	13.72	----	1.17×10^{-3}	45.45	12.74	-----
**0.1**	-1.499	6.44×10^{-4}	-0.245	0.290	7.02	48.9	5.9×10^{-4}	97.74	6.43	49.6
**0.2**	-1.482	5.57×10^{-4}	-0.255	0.218	6.07	55.8	5.00×10^{-4}	102.06	5.45	57.2
**0.5**	-1.463	1.86×10^{-4}	-0.256	0.260	2.03	85.2	1.97×10^{-4}	284.3	2.15	83.2
**1.0**	-1.449	7.18×10^{-5}	-0.273	0.257	0.78	94.3	7.61×10^{-5}	755.3	0.83	93.5
**2.0**	-1.435	4.03×10^{-5}	-0.265	0.202	0.44	96.8	4.80×10^{-5}	1036.9	0.52	95.9

Table S4 – Various electrochemical kinetic parameters recorded for compound **4 (the best one)**, derived from polarization measurements employing the Tafel extrapolation and LPR methods.

**[Inhibitor] / mM**	**Tafel extrapolation method**						**LPR method**			
	**E_{corr} / V *vs.* Ag/AgCl**	**j_{corr} / A cm^{-2}**	**β_c / mV dec^{-1}**	**β_a / mV dec^{-1}**	**υ / mm yr^{-1}**	***I*(%)**	**j_{corr} / A cm^{-2}**	**R_p / Ω cm^2**	**υ / mm yr^{-1}**	***I*(%)**
**0.0**	-1.520	1.26×10^{-3}	-0.249	0.241	13.72	----	1.17×10^{-3}	45.45	12.74	-----
**0.1**	-1.531	5.97×10^{-4}	-0.247	0.289	6.50	52.6	5.62×10^{-4}	102.90	6.12	52.0
**0.2**	-1.581	5.20×10^{-4}	-0.258	0.220	5.66	58.7	4.75×10^{-4}	108.55	5.17	59.4
**0.5**	-1.590	1.32×10^{-4}	-0.255	0.258	1.44	89.5	1.16×10^{-4}	480.05	1.26	90.1
**1.0**	-1.586	3.02×10^{-5}	-0.275	0.256	0.33	97.6	3.74×10^{-5}	1539.26	0.41	96.8
**2.0**	-1.567	1.39×10^{-5}	-0.268	0.199	0.15	98.9	1.87×10^{-5}	2651.77	0.20	98.4

S4. Cartesian coordinates for the optimized geometry of the studied xanthene:

(3)

C 0.62052000 -3.76771300 -0.21785500
C 2.14134700 -3.58836400 -0.00710400
C 2.39235900 -2.34100100 0.89289400
C 1.55127100 -1.19113900 0.46303700
C 0.41405300 -1.23807900 -0.25667300
C -0.06722700 -2.53859700 -0.78507900
O 2.11984900 0.00090800 0.91975900
C 1.55024500 1.19246600 0.46303700
C 0.41298800 1.23842700 -0.25667400
C -0.39599700 -0.00017500 -0.55908700
C 2.39034200 2.34305200 0.89289500
C 2.13825400 3.59020100 -0.00709800
C 0.61727200 3.76823700 -0.21785100
C -0.06941000 2.53853000 -0.78508200
C 2.70858300 -4.82910700 0.69974500
C 2.83943700 -3.39720100 -1.36135200
C 2.70441700 4.83143000 0.69975500
C 2.83651000 3.39964400 -1.36134600
C -1.67262600 -0.00072200 0.26799900
C -2.91908100 -0.00124500 -0.37599800
C -4.09813500 -0.00175000 0.38020100
C -4.03758800 -0.00173300 1.78293700
C -2.79766000 -0.00121300 2.42242000
C -1.61872200 -0.00070800 1.66786000
C -5.42177800 -0.00230600 -0.30457400
O -5.54622600 -0.00235600 -1.51025800
O -0.95010400 -2.63356900 -1.60687800
O -0.95236000 2.63274100 -1.60688900
H 0.12318800 -4.03170600 0.73745500
H 0.42726300 -4.63132300 -0.88753900
H 2.16459600 -2.58106800 1.95445700
H 3.46863600 -2.06911800 0.88468700
H -0.66248000 -0.00029000 -1.65515900
H 2.16237300 2.58291900 1.95446000
H 3.46685300 2.07209600 0.88468600
H 0.42327000 4.63168300 -0.88753000
H 0.11971100 4.03179500 0.73746100
H 2.22211300 -5.01721200 1.66248300
H 2.57263300 -5.73049400 0.08964300
H 3.78353200 -4.73141700 0.88745300
H 3.92483600 -3.30494500 -1.25304600
H 2.65287100 -4.24434500 -2.03218500
H 2.48493500 -2.49858800 -1.88108300
H 2.21778300 5.01911200 1.66249400
H 3.77945000 4.73466800 0.88746500
H 2.56769000 5.73270200 0.08965600
H 3.92198800 3.30832700 -1.25304000
H 2.48278500 2.50072700 -1.88108000
H 2.64921100 4.24662800 -2.03217700
H -2.98556500 -0.00126800 -1.47071300
H -4.95326800 -0.00212500 2.37225100
H -2.74525200 -0.00119800 3.50914600
H -0.65277400 -0.00030200 2.17122000
H -6.30188100 -0.00265400 0.36062500

(4)

C 0.38550500 3.55345200 -0.25201800
C -1.01130400 4.10695800 0.11301600
C -1.74092400 3.08839600 1.03981100
C -1.60277700 1.70044800 0.52299100
C -0.64380200 1.23099800 -0.29933600
C 0.35916400 2.16622100 -0.86249300
O -2.64688400 0.91185800 1.01064100
C -2.76401100 -0.37979700 0.49241000
C -1.84345000 -0.93506000 -0.31944600
C -0.56021800 -0.22868500 -0.67932500
C -4.02800200 -0.99905200 0.97414000
C -4.47788600 -2.16123300 0.03808900
C -3.25551700 -3.04416900 -0.30379300
C -2.09453600 -2.27466700 -0.90679300
C -5.52901500 -3.00728100 0.77235900
C -5.08261500 -1.58258200 -1.24916600
C 0.64436200 -0.85282400 0.01224100
C 1.88899100 -0.79747100 -0.63054300
C 3.02751700 -1.33880000 -0.01339400
C 2.91658400 -1.95897500 1.24251000
C 1.67427600 -2.01631600 1.87670600
C 0.54338900 -1.45977900 1.27161000
C 4.31211400 -1.25795600 -0.71912200
C 5.49374100 -0.90890100 -0.16247000
C -0.85219400 5.43503800 0.86801700
C -1.83696100 4.32881300 -1.16240900
O 1.12705500 1.85440700 -1.74795000
O -1.42488500 -2.74027300 -1.80078700
C 6.69688500 -0.86923100 -0.94446300
N 7.66353000 -0.84011000 -1.57713300
C 5.65837000 -0.53620900 1.21203600
N 5.79389100 -0.22934100 2.31825900
H 1.03716700 3.52140600 0.64623300
H 0.90039100 4.24761000 -0.94883100
H -1.32007900 3.13588600 2.06882300
H -2.80954400 3.36646300 1.15469700
H -0.41563600 -0.30932600 -1.79609700
H -3.87803800 -1.37508100 2.00994400
H -4.83354700 -0.23960100 1.06073600
H -3.55427600 -3.85389000 -1.00187100
H -2.89025000 -3.57092800 0.60096800
H -5.13380800 -3.45902300 1.68820000
H -6.40507400 -2.41120600 1.05153300
H -5.89141100 -3.82749600 0.14043500
H -5.97488800 -0.98089000 -1.04768800
H -4.37087300 -0.94276800 -1.78487000
H -5.38032200 -2.37624100 -1.94496800
H 1.96937800 -0.30165400 -1.60496300
H 3.79118300 -2.40035300 1.71886400
H 1.58794800 -2.49445300 2.85194000
H -0.41710400 -1.50118500 1.78246000
H 4.26478300 -1.49490500 -1.79384700
H -0.25142800 5.32705100 1.77746700
H -0.35739000 6.19039000 0.24510600
H -1.82166800 5.85202700 1.16268200
H -2.82279000 4.75163900 -0.94284600
H -1.33596000 5.02153300 -1.84928500
H -1.99710600 3.39517100 -1.71491600

(5)

C -0.04506200 3.60182900 -0.24824600
C -1.47529200 4.10817400 0.04957100
C -2.20076500 3.08264700 0.97189800
C -1.99183400 1.69045900 0.49142800
C -0.98540000 1.24102800 -0.28401200
C 0.00715900 2.20053300 -0.82423900
O -3.02352700 0.87346900 0.95754800
C -3.07165900 -0.43351900 0.46716000
C -2.10222700 -0.96991700 -0.29898400
C -0.83712800 -0.22063800 -0.63562900
C -4.32653400 -1.09144000 0.92049600
C -4.69804500 -2.29115700 -0.00262200
C -3.43125800 -3.13274800 -0.28092600
C -2.27970800 -2.33232300 -0.86104500
C -5.74111400 -3.16108600 0.71499600
C -5.27867900 -1.76578800 -1.32332800
C 0.36965200 -0.78840900 0.09880800
C 1.62452000 -0.72535300 -0.52308000
C 2.76481400 -1.21356000 0.13494500
C 2.64434000 -1.78981700 1.41122700
C 1.39192400 -1.85753600 2.02358200
C 0.25961300 -1.35215500 1.37726600
C 4.05835900 -1.12635400 -0.54997000
C 5.22896100 -0.73325200 0.00559300
C -1.39703100 5.45803200 0.77825000
C -2.25820600 4.26890300 -1.26138100
O 0.82708300 1.89716800 -1.66475100
O -1.55976600 -2.79167800 -1.71823400
C 6.45559400 -0.72406600 -0.85066800
O 6.55377900 -1.25202000 -1.93012400
O 7.56528600 -0.06001300 -0.39057500
C 5.35356600 -0.32466700 1.36371800
N 5.46132700 0.01416000 2.46500800
H 0.57198900 3.61737100 0.67438200
H 0.46890100 4.29912200 -0.94273200
H -1.82142800 3.16813200 2.01438300
H -3.28268700 3.32213300 1.04067400
H -0.65915300 -0.31354900 -1.74667000
H -4.19840200 -1.43813600 1.96934800
H -5.16356000 -0.36280400 0.96156900
H -3.67365700 -3.96873400 -0.97001200
H -3.07873500 -3.62516400 0.64791000
H -5.36158100 -3.57623000 1.65447200
H -6.64893400 -2.59390300 0.94928600
H -6.04897600 -4.00902300 0.09071200
H -6.19990300 -1.19504200 -1.16718600
H -4.57353300 -1.11105800 -1.84963300
H -5.52114600 -2.58603900 -2.00974900
H 1.71099800 -0.26372500 -1.51399000
H 3.52071900 -2.19179100 1.91896700
H 1.29823000 -2.30327900 3.01338600
H -0.70992700 -1.40001900 1.87081100
H 4.04174700 -1.40130900 -1.62194300
H -0.82935100 5.39414900 1.71269300
H -0.90728400 6.21803600 0.15697900
H -2.39274000 5.84331700 1.02482000
H -3.26776300 4.65656100 -1.08988400
H -1.75822800 4.96598200 -1.94462100
H -2.35985700 3.31781300 -1.79782400
H 7.48077300 0.39577100 0.47904300

(6)

C -3.13593200 -3.43107000 0.70300900
C -4.58150500 -2.89270000 0.59803200
C -4.62099600 -1.71327000 -0.42063700
C -3.47817300 -0.78374500 -0.21336300
C -2.31239100 -1.05077500 0.40530600
C -2.11618800 -2.36706500 1.06451600
O -3.78081800 0.46377500 -0.76746700
C -2.88330300 1.50338500 -0.52027600
C -1.69502900 1.32655500 0.09064200
C -1.19180700 -0.04414100 0.47787200
C -3.45182900 2.78471300 -1.01894800
C -2.81672600 4.00267100 -0.28325100
C -1.28340100 3.81480100 -0.21691000
C -0.84782900 2.50101600 0.40374100
C -3.14039300 5.27993600 -1.07307000
C -3.39384900 4.10827400 1.13568200
C -0.03552800 -0.42464000 -0.43631900
C 1.27131000 -0.13011600 -0.02634800
C 2.36395200 -0.46732400 -0.84344400
C 2.14033400 -1.07750200 -2.08895800
C 0.83510700 -1.35345900 -2.50176600
C -0.24989200 -1.03239500 -1.68141600
C 3.71193900 -0.13771000 -0.36788700
C 4.82045600 -0.89905500 -0.50187500
C -5.50675800 -4.01067600 0.09411500
C -5.05873000 -2.40248600 1.97279900
O 0.14400700 2.43460100 1.09835300
O -1.21228800 -2.59006200 1.83673800
C 6.14028700 -0.45697700 0.03422000
O 7.15415300 -1.09767000 0.16303000
O 6.06829300 0.87641600 0.37996600
C 7.35488600 1.07756700 2.43977800
C 7.25473000 1.48602400 0.98601000
C 4.84068400 -2.18386500 -1.12671800
N 4.85786000 -3.22314600 -1.63359700
H -2.82408500 -3.90177200 -0.25148400
H -3.08696800 -4.24938600 1.45144000
H -4.58772100 -2.10359400 -1.46153300
H -5.58888300 -1.17403600 -0.34877600
H -0.81733700 -0.00997000 1.54210800
H -3.27212400 2.86031600 -2.11412800
H -4.55648500 2.79907000 -0.90856100
H -0.82118600 4.65251500 0.34571900
H -0.84081200 3.87777600 -1.23257500
H -2.73087200 5.25596900 -2.08842600
H -4.22111500 5.43940500 -1.15876200
H -2.72624600 6.16820500 -0.58051400
H -4.47627600 4.27293100 1.12562100
H -3.20697000 3.20076300 1.72259900
H -2.94678400 4.94196100 1.69015300
H 1.43246900 0.37325300 0.93306700
H 2.97816300 -1.32844100 -2.73930900
H 0.66423000 -1.82676100 -3.46804900
H -1.26292000 -1.25448400 -2.01233700
H 3.79205600 0.83109800 0.16010600
H -5.18865500 -4.40819000 -0.87542100
H -5.52845600 -4.85456200 0.79476600
H -6.53981500 -3.66265700 -0.01581300
H -6.09561800 -2.05229700 1.94523500
H -5.00849100 -3.20097100 2.72293400
H -4.44379200 -1.57604500 2.34916800
H 8.14065000 1.63693200 2.96114600
H 6.41149600 1.24770700 2.97481500
H 7.59378100 0.00720700 2.53773700
H 8.14088900 1.18948300 0.39661500
H 7.02552300 2.55826800 0.84545500
